# Worth the paper it’s written on? A cross-sectional study of Medical Certificate of Stillbirth accuracy in the UK

**DOI:** 10.1093/ije/dyac100

**Published:** 2022-06-20

**Authors:** Michael P Rimmer, Ian Henderson, William Parry-Smith, Olivia Raglan, Jennifer Tamblyn, Alexander E P Heazell, Lucy E Higgins, the UKARCOG NESTT working group authors, H Aadan, K F Ajoku, S Asim, E J Badger, L E Barfi, L M Bevington, M Bhat, N Black, R E Black, S A Boughey, C B Brewster, C E Buchanan, S H Bullough, V Byrne, C C Carpenter, S A Carron, F Conti-Ramsden, V C Cordell, S Craggs, L C Creswell, A Cury Fernandes, J A Dalton, D V Dracocardos, C E Dunlop, J K Egan, C I Ejiofor, C H J Elderfield, D Faluyi, D M Geddes-Barton, T Giacchino, S E Giles, E A Goodier, J K Goodman, M Govil, R Grainger, E A Guyett, A J Haken, R C Harrison, J L Hartley, F S Hogg, E Hutchinson, H S Jamie, L A C Jamison, S Jaufuraully, G Jethwani, C M Jones, I Karapanos, B Karavadra, L S Kasaven, R Kaur, A J Kermack, A King, C L B Lallemant, K R Lattey, E A Layden, C A MacMahon, L R Maddy, S M Magee, L Mahilchi Sudar, A Mahmud, K J Marks, A McNally, E S Medford, S V I Milliken, O M Mogekwu, H E Mohamed, S S Moorhouse, M Mouhajer, H Mumtaz, L L Murphy, K Navaratnam, A E Neville, S K Nijjar, S O’Brien, M H Obeysekera, R I Odonde, O Ofodile, N Okagbue, L Parnell, M D Pearce, M Petrovic, S R Picart, C L Plant, H M Powell, S E Powell, L Preston, O Raglan, M Ramcharn, K M Reilly, N Riaz, J M Riches, D R Rutherford, P Sathyendran, F Shamsudin, M Simonian, K E Smith, J K Sohal-Burnside, L J Standing, L I Stirrat, L J Stocker, K Subba, C Summerhill, C J Taylor, S Thomson, K C A Thyne, F Tomlinson, J Troko, N Verasingam, H E Welch, K M Whittle, S G Williams, K J Wilson, S F Wilson, W P Wilson-Theaker, C L M Wyeth

**Affiliations:** United Kingdom Audit and Research Collaborative in Obstetrics and Gynaecology, UK; MRC Centre for Reproductive Health, Queens Medical Research Institute, Edinburgh BioQuarter, Edinburgh, UK; United Kingdom Audit and Research Collaborative in Obstetrics and Gynaecology, UK; Warwick Medical School, University of Warwick, Coventry, UK; United Kingdom Audit and Research Collaborative in Obstetrics and Gynaecology, UK; Department of Obstetrics and Gynaecology, Shrewsbury and Telford NHS Trust, Apley, UK; United Kingdom Audit and Research Collaborative in Obstetrics and Gynaecology, UK; Department of Obstetrics and Gynaecology, Chelsea and Westminster NHS Trust, London, UK; United Kingdom Audit and Research Collaborative in Obstetrics and Gynaecology, UK; Institute of Metabolism and Systems Research, University of Birmingham, Birmingham, UK; Department of Reproductive Medicine, Seacroft Hospital, Leeds, UK; Maternal and Fetal Health Research Centre, University of Manchester, Manchester, UK; United Kingdom Audit and Research Collaborative in Obstetrics and Gynaecology, UK; Maternal and Fetal Health Research Centre, University of Manchester, Manchester, UK

**Keywords:** Stillbirth, perinatal death, cause of death, fetal growth restriction, placental insufficiency, accuracy, death certification, stillbirth certification

## Abstract

**Background:**

The Medical Certificate of Stillbirth (MCS) records data about a baby’s death after 24 weeks of gestation but before birth. Major errors that could alter interpretation of the MCS were widespread in two UK-based regional studies.

**Methods:**

A multicentre evaluation was conducted, examining MCS issued 1 January 2018 to 31 December 2018 in 76 UK obstetric units. A systematic case-note review of stillbirths was conducted by Obstetric and Gynaecology trainees, generating individual ‘ideal MCSs’ and comparing these to the actual MCS issued. Anonymized central data analysis described rates and types of error, agreement and factors associated with major errors.

**Results:**

There were 1120 MCSs suitable for assessment, with 126 additional submitted data sets unsuitable for accuracy analysis (total 1246 cases). Gestational age demonstrated ‘substantial’ agreement [K = 0.73 (95% CI 0.70–0.76)]. Primary cause of death (COD) showed ‘fair’ agreement [K = 0.26 (95% CI 0.24–0.29)]. Major errors [696/1120; 62.1% (95% CI 59.3–64.9%)] included certificates issued for fetal demise at <24 weeks’ gestation [23/696; 3.3% (95% CI 2.2–4.9%)] or neonatal death [2/696; 0.3% (95% CI 0.1–1.1%)] or incorrect primary COD [667/696; 95.8% (95% CI 94.1–97.1%)]. Of 540/1246 [43.3% (95% CI 40.6–46.1%)] ‘unexplained’ stillbirths, only 119/540 [22.0% (95% CI 18.8–25.7%)] remained unexplained; the majority were redesignated as either fetal growth restriction [FGR: 195/540; 36.1% (95% CI 32.2–40.3%)] or placental insufficiency [184/540; 34.1% (95% CI 30.2–38.2)]. Overall, FGR [306/1246; 24.6% (95% CI 22.3–27.0%)] was the leading primary COD after review, yet only 53/306 [17.3% (95% CI 13.5–22.1%)] FGR cases were originally attributed correctly.

**Conclusion:**

This study demonstrates widespread major errors in MCS completion across the UK. MCS should only be completed following structured case-note review, with particular attention on the fetal growth trajectory.

Key Messages

- Almost 80% of Medical Certificates of Stillbirth in the UK contained errors; 55.9% had a major error that would alter MCS interpretation.
- 43.3% of all stillbirths were officially registered as being of ‘unknown cause of death’ (COD); 78% of these had an identifiable primary COD.
- Fetal growth restriction (FGR) was the leading primary COD (24.6%); many such deaths may have been preventable.
- With basic guidance, non-expert reporters can redress one of these core errors: converting ‘unexplained’ to explained deaths (principally FGR and placental conditions).
- This will improve both the accuracy of stillbirth cause data, and also outcomes for families and babies.

## Introduction

Stillbirth is defined in the UK as a child born after 24 weeks’ gestation without signs of life,^1^ occurring in 3.9/1000 English and Welsh births in 2018.^2^ In the Medical Certificate of Stillbirth (MCS), the issuer records the probable cause of death (COD); this is issued to parents to legally register their child’s death. The hospital retains a ‘counterfoil’ summarizing the information recorded on the corresponding MCS. Whereas stillbirth registration can be deferred for 21, 42 or 365 days in Scotland, England/Wales and Northern Ireland, respectively, in practice registration is required prior to the body being buried or cremated. Therefore, the MCS is traditionally issued within days.^3^

MCS data are collated nationally to report on stillbirth incidence, timing and cause.^4^^,^^5^ An ‘unexplained’ MCS COD is legally acceptable in the UK,^6^ with no external Coronial accuracy checks.^7^ Two regional UK-based studies examined MCSs issued in North-West England during 2009 and 2015^8^^,^^9^ comprising 213/3688 (5.8%) and 243/3147 (7.7%) of reported stillbirths in England and Wales during the study periods.^2^ It was found that 74.2% and 53.6% of 2009 and 2015 stillbirths, respectively, were registered as ‘unexplained’ but only 18.3% and 13.9% remained unexplained after review (cases reviewed for adjudicated COD comprised 93.0% and 91.4% of total cases submitted, respectively). Major errors (those likely to affect MCS interpretation by family, healthcare professionals or statisticians^10^) were found in 49% of MCSs in 2015; minor errors (those that do not affect MCS interpretation^10^) were found in a further 25%.

Accurately recording stillbirth COD is important for families and local and national learning. The MCS is the first and often only information source for bereaved parents and future caregivers to guide future pregnancy or maternal health management,^11^ as only a minority undergo post-mortem investigation.^12^ Studies in adult death certification suggest that certifier profession and training may affect accuracy.^13^^,^^14^ Data from MCSs also inform healthcare service strategy, funding, research and public health initiatives.^5^ It is imperative to identify preventable stillbirths to aid future strategies to reduce deaths. This will support international governments in meeting the Every Newborn Action Plan 2030 targets.^15^

We hypothesized that (i) the previous finding from regional studies, that a majority of MCSs have inaccuracies, would be replicated nationwide; (ii) major MCS error would be related to issuer profession and seniority.

## Methods

The study is reported in accordance with STROBE guidelines (Supplementary Table S1, available as Supplementary data at *IJE* online). A total population sampling method was adopted.

The United Kingdom Audit and Research Collaborative in Obstetrics and Gynaecology (UKARCOG),^16^ a trainee-led organization, maintains regional representatives in all seven National Health Service Health Education England regions^17^ and one representative each in Northern Ireland, Scotland and Wales. Representatives recruit local data collectors (doctors pursuing a career in obstetrics and gynaecology) to specific project working groups from every hospital in their region. For this project, no restrictions were placed on local data collectors’ seniority/prior training. Local data collectors registered the individual project with their local governance processes.

Cases were identified via birth registers, MCS counterfoils and local perinatal mortality registers. In previous regional studies >90% of cases were identified via local perinatal mortality registers and MCS data were obtained from counterfoils in >75% of cases.^8^^,^^9^ Inclusion criteria were birth during the study period of 1 January 2018 to 31 December 2018 and (i) MCS issuance or (ii) where an MCS should have been issued but was not.^18^ In accordance with UK law, stillbirth following medical termination of pregnancy beyond 24 completed weeks’ gestation was included. Potential cases were excluded if local data collectors were unable to verify eligibility for legal registration of birth (whether stillborn or live-born) against maternal medical records or in late miscarriage/neonatal death cases where no evidence of an MCS actually being issued was found. The 1-year study period was chosen to mitigate temporal changes in COD and workforce experience that may bias the study findings and for a realistic workload (estimated 2000–2500 cases based on national stillbirth rates and assuming data submitted from 80% of UK maternity units).^2^^,^^19^ Data collection commenced in August 2019 after junior doctor hospital rotations, minimizing potential for local data collectors to self-audit.

### Data collection

An electronic local data collection tool (Supplementary File S2, available as Supplementary data at *IJE* online) was adapted from previous studies,^8^^,^^9^ piloted using case notes from one hospital (L.H.) and tested with fictional data combinations (M.R. and W.P.S.). Contemporaneous parental autopsy wishes were incompletely documented in maternal medical records, preventing accurate assessment against MCSs, and MCS autopsy status accuracy was therefore excluded from review. Local data collectors received written instructions for structured case review^20^ and data collection tool use.

**Figure 1:**
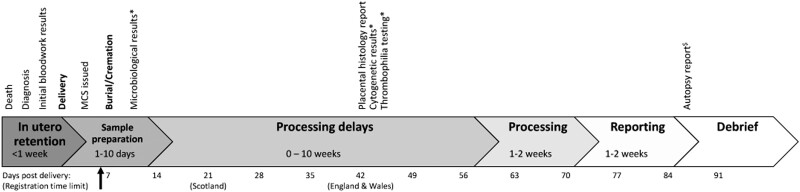
Typical timeline for reporting post-mortem investigation results in the UK. Reporting timeline based on full uptake of all recommended investigations,^11^ including autopsy, and average reporting times;^3^ days since delivery are shown along the bottom with territory-specific legal registration deadlines in brackets [Northern Ireland deadline for registration (365 days) not pictured in displayed timeline]. In practice, legal registration of stillbirth must be complete before burial or cremation; therefore, it is commonly completed within a week of delivery (indicated by bold arrow). The National Health Service aims to report 60% of autopsy investigations within 42 days of examination and 90% within 56 days, longer where specialist opinions are required.^22^ Accounting for processing time and delays, many autopsy examinations can take up to 12 weeks for reporting; placental histopathology without autopsy is often reported by 6 weeks post-delivery. Similar timescales are anticipated in other international healthcare settings. In practice the true time from death to delivery is unknown and may, particularly in cases of co-twin demise, take several weeks. However, diagnosis-to-delivery intervals of >1 week are unusual in singleton pregnancies. * indicates investigations that are not always indicated. Autopsy consent is optional (^$^) and not required under Coronial jurisdiction in the UK.

Data regarding the woman’s background, pregnancy health prior to fetal death, care provided and information documented on the actual MCS were reviewed locally, limited to data available at MCS completion (contemporaneous data; Figure 1).^3^^,^^11^^,^^21^ Delayed investigation results, defined as those not reported prior to the MCS being issued (including autopsy, placental histopathology, cytogenetics, thrombophilia screening and some microbiological tests), were disregarded unless reported prior to MCS completion, as these results could not have been known by the MCS issuer. An ‘ideal MCS’ was constructed by the data collector. Documented and ideal CODs were categorized according to the Relevant Condition at Death (ReCoDe) classification system (Table 1).^22^ Conditions present but not contributing to death were deemed ‘associated’ rather than ‘causal’. Where multiple conditions were considered causal, their hierarchy (direct or indirect) was determined according to likely contribution to the infant’s demise. Local data collectors assessed the accuracy (‘accurate’, ‘inaccurate’ or ‘unknown’) of MCS-reported infant sex, delivery date, birthweight, timing in relation to labour onset (termed ‘accuracy data’).

**Table 1:** Suggested use of ReCoDe classification system for cause of death in completion of Medical Certificates of Stillbirth

Category	Subcategory	Usually	Consider
Fetus	Lethal congenital anomaly Infection: 2.1 Chronic, 2.2 Acute Non-immune hydrops Iso-immunization Fetomaternal haemorrhage Twin–twin transfusion Fetal growth restriction	Fetal direct (a)	Fetal indirect (b) OR Other contributory (e)
	8. Fetal (other)		Fetal direct (a) OR Fetal indirect (b) OR Other contributory (e)
B. Umbilical cord	Prolapse Constricting loop or knot	Fetal direct (a)	
	3. Velamentous insertion		Fetal indirect (b)
	4. Umbilical cord (other)		Fetal direct (a) OR Fetal indirect (b) OR Other contributory (e)
C. Placenta	Abruption Praevia Vasa praevia	Fetal direct (a)	
	4. Placental insufficiency/infarction	Fetal direct (a) OR Fetal indirect (b)	
	5. Placental (other)		Fetal direct (a) OR Fetal indirect (b)
D. Amniotic fluid	Chorioamnionitis Oligohydramnios Polyhydramnios	Fetal direct (a) OR Fetal indirect (b)	
	4. Amniotic fluid (other)		Fetal direct (a) OR Fetal indirect (b) OR Other contributory (e)
E. Uterus	Rupture	Maternal direct (c)	
	2. Uterine (other)		Maternal direct (c) OR Maternal indirect (d) OR Other contributory (e)
F. Mother	Diabetes Thyroid disease Essential hypertension Hypertensive disease in pregnancy Lupus/antiphospholipid syndrome Cholestasis Drug abuse		Maternal direct (c) OR Maternal indirect (d) OR Other contributory (e)
	8. Maternal (other)		Maternal direct (c) OR Maternal indirect (d) OR Other contributory
G. Intrapartum	Asphyxia Birth trauma	Fetal direct (a)	
H. Trauma	External Iatrogenic^a^	Fetal direct (a)	Maternal direct (c) OR Maternal indirect (d)
I. Unclassified	No relevant condition identified No information available	Fetal direct (a)^b^	

With reference to standard UK Medical Certificates of Stillbirth (MCSs) including fetal [direct (a) and indirect (b)], maternal [direct (c) and indirect (d); in Northern Ireland maternal causes are not subclassified as direct or indirect] and associated or contributory (e) fields. The authors recommend that where indicated, a condition would be reported in the MCS field recommended under ‘usually’, unless the assessor considers that there is concurrent evidence of a more or less significant causative role in the infant’s death. For example, in the case of a growth-restricted infant with evidence of massive placental abruption where the infant was known or suspected to have been alive at the time of abruption and demised after this event, fetal growth restriction would be recorded as either fetal indirect (b) or other contributory (e) according to reviewer assessment of the contribution of the growth restriction to the infant’s demise.

a Iatrogenic causes of stillbirth include termination of pregnancy resulting in the stillbirth of an infant known to have been alive after 24 completed weeks of pregnancy, in accordance with UK law.

b Unclassified designations of cause of death should not be included on a MCS where another potentially relevant cause [(a)–(d)] has been identified. Table adapted from Higgins *et al*.,^9^ copyright Wiley-Blackwell Open Online.

As in previous regional studies the following definitions were applied: lethal fetal abnormality (abnormality typically associated with ≥50% perinatal mortality), late miscarriage (fetal death *in utero* or birth without sign of life between 18^+0^ and 23^+6^ weeks’ gestation) and placental insufficiency (placental weight <10th centile for gestation and fetal sex,^23^ abnormal umbilical artery Doppler waveform, oligohydramnios without fetal abnormality or ruptured membranes, or documented small, infarcted, gritty or otherwise abnormal placenta at birth).^9^ Oligohydramnios, abnormal umbilical artery Doppler, abnormal other Doppler (including but not limited to uterine artery, middle cerebral artery, Ductus venosus) and other conditions were defined according to local policy. ‘Previous audit region’ was hospitals included in prior North-West England regional audits of MCS accuracy.

Customized estimated fetal weight and individualized birthweight centiles were calculated using the Bulk Centile Calculator V.8.0.4 (Perinatal Institute, Birmingham, UK). In order to reduce potential for fetal growth restriction (FGR) over-diagnosis, gestation calculated from the date of fetal death *in utero* confirmation was used to calculate the birthweight centile, with an in-built 2-day gestation ‘correction’ within the centile calculator where fetal demise is indicated. FGR was defined according to the 2016 live-born FGR consensus definition with an additional criterion: loss of >50 centiles in symphysio-fundal height (with no growth scans), provided birthweight <50th centile.^24^ Abdominal circumference centiles were calculated according to Chitty *et al.*^25^

The completing clinician seniority was categorized as junior (Registered Midwife Band 5/6, Obstetrics and Gynaecology speciality trainee Years 1–5 or equivalent) or senior (Registered Midwife Band 7+, Obstetrics and Gynaecology speciality trainee Years 6+, Consultant Obstetrician or equivalent).

### Data review

Several quality-control measures were taken. Data collection form validation rules alerted local data collectors contemporaneously when inconsistent/impossible data were entered (e.g. date, gestation, age, weight or height outside plausible range). Summary data, after personal identifying data were removed, were analysed centrally for eligibility by two independent reviewers (L.H. and M.P.R.) who did not participate in data collection. Cases were excluded if eligibility could not be assured, e.g. simultaneously reporting no access to MCS data (therefore lacking MCS issuance evidence) and either unknown gestation with birthweight <500 g or possible signs of life at birth for the same case.^1^

Missing values were not imputed. Where cases were excluded from accuracy assessment (all accuracy assessment data missing), included and excluded case characteristics were compared (Mann–Whitney U test and Chi^2^ test). Where some (but not all) accuracy data were reported, cases were retained and missing data were assumed to be accurate. Local data collector assessments could not be validated against post-mortem findings (low uptake) or national perinatal mortality review findings (case/hospital anonymity), but have previously been shown to be similar.^9^^,^^26^

### Data analysis

Primary analyses were major and minor error rates (defined as in Table 2), error-free rates, and primary COD and gestational age accuracy [Cohen’s kappa coefficient (Κ)].^27^ Potentially identifiable data (including indirectly identifying data combinations such as date and timing in relation to delivery) were not submitted centrally. MCS counterfoils do not include infant sex. Thus preventing other accuracy field chance-adjusted agreements being calculated.

**Table 2:** Definitions of major and minor errors in Medical Certificate of Stillbirth completion used in the study

	Major error	Minor error
Definition	Materially affecting interpretation of MCS by family, healthcare professionals or healthcare statisticians	Inaccuracies falling short of definition of major error
Standard criteria	MCS issued where infant demised after <24 completed weeks’ gestation even if delivery occurring after the date on which 24 weeks’ gestation would have been achieved	Incorrect date of delivery
		Incorrect sex of infant
		Incorrect birthweight^a^
	MCS issued where infant showed signs of life at birth	Incorrect gestation^b^
	Incorrect primary cause of death on MCS	Incorrect timing in relation to labour^b^

Definitions were based on the original definition of adult death certificate errors described by Pritt *et al*.^10^ and adapted for Medical Certificates of Stillbirth (MCS) as previously by Cockerill *et al.*^8^ and Higgins *et al*.^9^

a Errors in birthweight were not considered to be major, as if this resulted in failure to document the contribution of abnormal fetal growth, this would be covered under either incorrect primary cause of death (major error) or inclusion/exclusion of a contributory condition (not) present (minor error). Comparisons of accuracy were absolute; the magnitude of difference between true and documented values did not affect classification as a major or minor error, as these variables would not affect the interpretation of the certificate, except where indicated

b in which case if the requirements for major error were met, this would be classified as a major error.

Major MCS error odds were calculated for issuer profession and seniority, using multilevel regression adjusting for clustering within hospitals. Based on previous regional studies, we estimated 99% power to detect a major error association of magnitude odds ratio (OR) <0.67 when comparing doctors to midwives, with 50% precision and 1:3 ‘exposure’ to ‘non-exposure’; power for other exposure/outcome pairs will vary. Exploratory secondary analyses compared individual condition/category incidences between actual and ideal MCS and major error association with maternal, healthcare and case characteristics but lack power to detect subtle relationships with lower exposure frequencies. Sensitivity analysis (complete records only) was conducted.

## Results

### Study cohort

**Figure 2:**
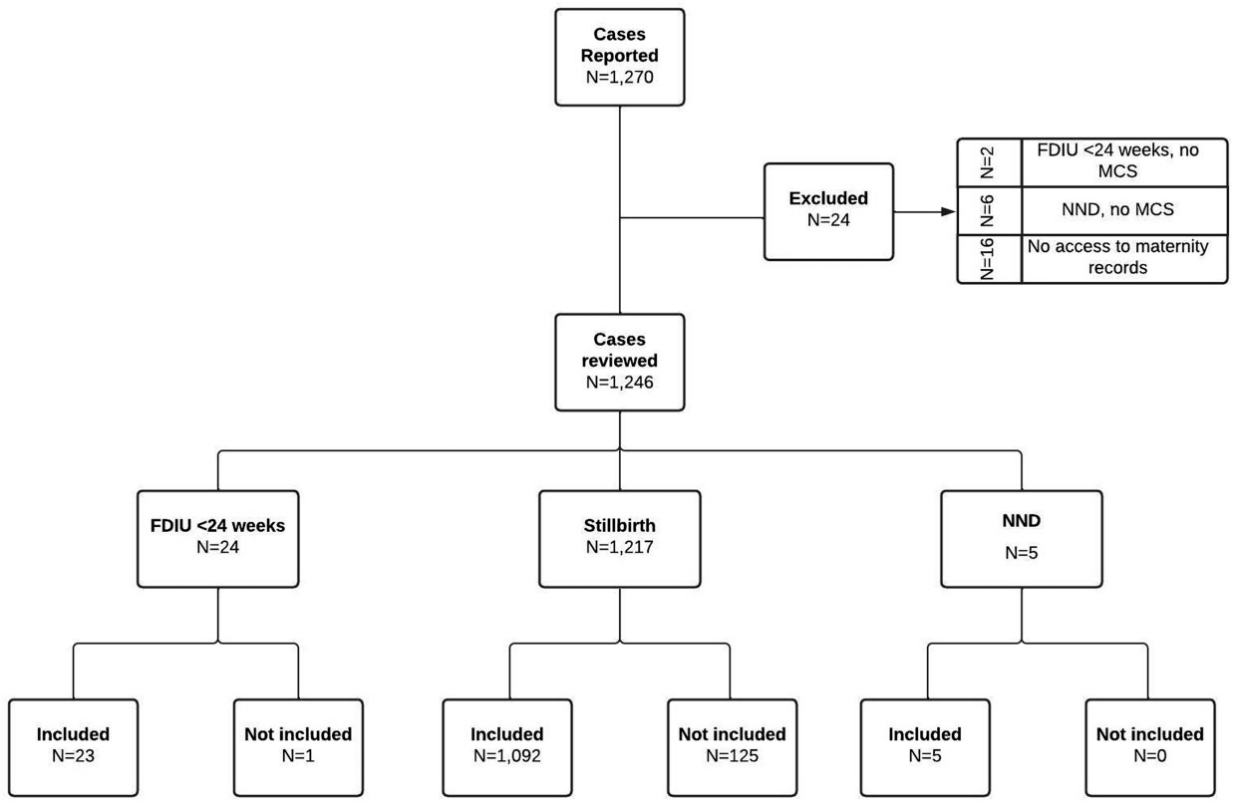
Flow of cases through the study. Cases were assessed against study inclusion and exclusion criteria [all births where a Medical Certificate of Stillbirth (MCS) was issued or should have been issued including cases where an MCS was issued in error including death prior to 24 weeks’ gestation or neonatal deaths]. Cases were included/not included according to whether at least some/no accuracy data were reported. FDIU, fetal death *in utero*; MCS, Medical Certificate of Stillbirth (the document issued by registered medical or midwifery practitioners to the parents of a stillborn infant for the purposes of legally registering the stillbirth); NND, neonatal death (death after being born with signs of life, even if not capable of supporting life).

The UKARCOG National Evaluation of Accuracy of Stillbirth Certificates (NESTT) working group comprised 115 individuals from 76/158 (49.4%) UK healthcare trusts providing maternity services in 2018 (including data submission from at least one hospital from all 10 UKARCOG representative regions). Of 1270 cases submitted, 24 (1.9%) were excluded and 126 (9.9%) were unsuitable for accuracy analysis due to non-submission of any MCS accuracy data (Figure 2); 866/1120 (77.3%) of cases reported MCS data according to the counterfoil summary. No identified registerable stillbirths lacked an issued MCS; 28 MCSs were issued to infants who were not stillborn. Missingness of accuracy data was as follows: infant sex (1010/1246; 81.1%), delivery date (327/1246; 26.2%), gestation (255/1246; 20.5%), birthweight (249/1246; 20.0%), timing in relation to labour onset (275/1246; 22.1%) and relevant conditions at death (188/1246; 15.1%); 1120 cases were included in the MCS accuracy analysis, representing 38.2% of 2932 stillbirths nationally in 2018. ^28–30^Supplementary Table S3 (available as Supplementary data at *IJE* online) summarizes data collected. Cases assessed for MCS accuracy differed from those excluded by country (*P *<* *0.0001) and level of maternity care provided in the hospital issuing the MCS (*P *=* *0.032), initial level of maternity care provided (*P *=* *0.011), antenatal assessment of fetal growth (*P *=* *0.014), birthweight centile (*P *=* *0.027) and diagnosis-to-delivery interval (*P *=* *0.006) (Supplementary Table S3, available as Supplementary data at *IJE* online).

### Primary analyses

**Figure 3:**
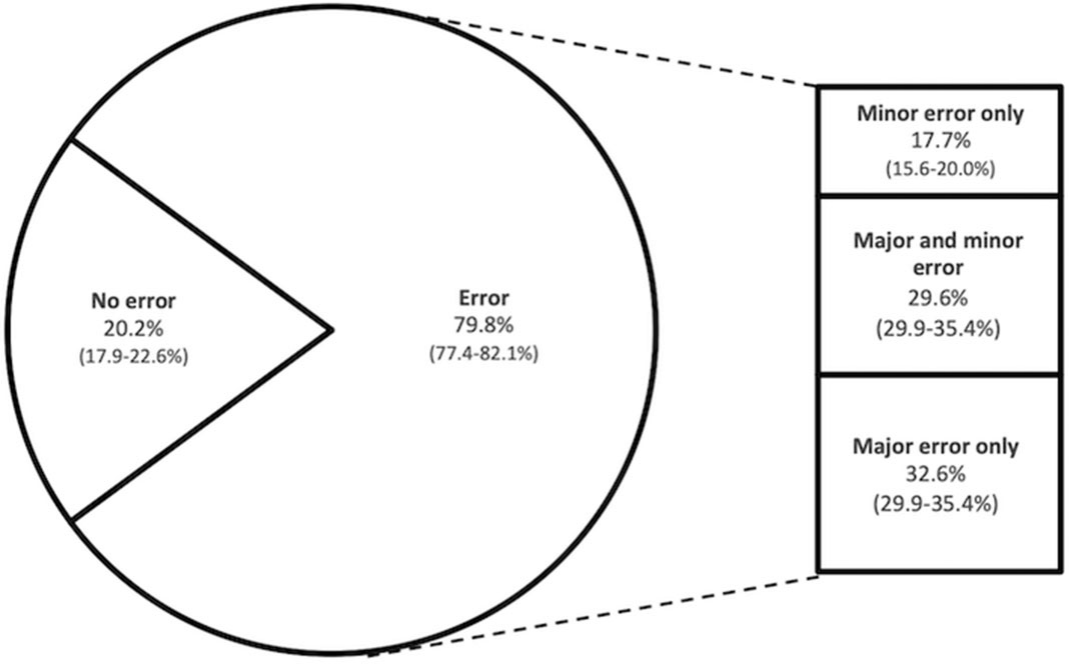
Incidence and grade of error categories in Medical Certificates of Stillbirth (*N* = 1120). Of all Medical Certificates of Stillbirth (MCSs) that could be assessed for accuracy, 79.8% contained at least one error; 29.6% of MCSs contained compound errors. Major errors (defined as those that would materially affect the interpretation of the certificate by the family, healthcare professionals and healthcare statisticians) were present in 62.2% of MCSs. These comprised (i) MCS issued pertaining to an infant proven to have demised prior to 24 completed weeks' gestation or an infant born with signs of life (regardless of gestation or whether capable of sustaining life), (ii) incorrect primary cause of death on MCS. Minor errors (defined as errors without significant impact on interpretation of the MCS) occurred in 47.3% of MCSs. These comprised inaccuracies in documented (i) sex, (ii) date of delivery, (iii) gestational age (falling short of major error), (iv) birthweight or (v) timing in relation to the onset of labour. Data are presented as numbers and expressed as a percentage (with 95% CI) of the proportion of cases where accuracy could be assessed. Certificates containing errors are subdivided in the right-hand bar according to error category.

In total, 81.9% (95% CI 79.6–83.9%) MCSs contained at least one major or minor error; 26.6% (95% CI 24.2–29.1%) contained both major and minor errors (Figure 3) and 668/696 (96.0%; 95% CI 94.3–97.2%) major errors resulted from incorrect primary COD assignment. The most common minor error was inaccurately documented gestational age (25% of cases in which gestation accuracy was assessed); this, and other minor error types, did not differ between MCSs with and without major error (Table 3). Non-primary contributory conditions were omitted from MCSs in 701/1032 (67.9%; 95% CI 65–70.7%) cases and non-contributory conditions were incorrectly attributed as causal in 208/1032 (20.2%; 95% CI 17.8–22.7%) cases. Errors in maternal or fetal attribution of causal conditions were noted in a further 51/1032 (4.9%; 95% CI 3.8–6.4%).

**Table 3:** Frequency of different Medical Certificate of Stillbirth error types by major error status

Error	Overall (*N* = 1120)	Major error absent/unknown (*N* = 550)	Major error present (*N* = 696)
**Major errors**			
Inaccurate primary cause of death	668/1032 (64.7%; 61.8–67.6%)	N/A	668/696 (96.0%; 94.3–97.2%)
Fetal death <24 weeks’ gestation	23/991 (2.3%; 1.6–3.5%)	N/A	23/696 (3.3%; 2.2–4.9%)
Neonatal death	5/971 (0.5%; 0.2–1.2%)	N/A	5/696 (0.7%; 0.3–1.7%)
**Minor errors**			
Sex	3/236 (1.3%; 0.4–3.7%)	1/78 (1.3%; 0.2–6.9%)	2/158 (1.3%; 0.4–4.5%)
Date of birth	103/919 (11.2%; 9.3–13.4%)	39/354 (11.0%; 8.2–14.7%)	64/565 (11.3%; 9.0–14.2%)
Gestation	251/991 (25.3%; 22.7–28.1%)	88/362 (24.3%; 20.2–29.0%)	163/629 (25.9%; 22.6–29.5%)
Birthweight	67/997 (6.7%; 5.3–8.5%)	25/308 (8.1%; 5.6–11.7%)	42/689 (6.2%; 4.7–8.3%)
Timing in relation to labour	214/971 (22.0%; 19.6–24.8%)	88/365 (24.1%; 20.0–28.8%)	126/606 (20.8%; 17.8–24.2%)

Data are presented as number of cases/denominator and expressed as percentage with 95% CIs. Data completeness was suboptimal for certain data fields, e.g. fetal sex where the data collector only had access to the Medical Certificate of Stillbirth counterfoil (which does not record infant sex); denominators were therefore adjusted to account for records with missing data.

After adjustment for agreement by chance, agreement between MCS documented gestational age and the gestational age at fetal death *in utero* confirmation was ‘substantial’ (Κ = 0.73; 95% CI 0.70–0.76). Corresponding agreement for primary COD was ‘fair’ (Κ = 0.26; 95% CI 0.24–0.29). Factors associated with major MCS error are shown in Table 4; the MCS issuer profession and seniority were unrelated to major error odds (*P *>* *0.05).

**Table 4:** Adjusted odds of major errors in Medical Certificates of Stillbirth: hypothesis testing and exploratory variables

	Whole cohort (*N* = 1120)			Sensitivity cohort (*N* = 181)		
	Major errors per category/total records in category	Odds ratio	95% CI	Major errors per category/total records in category	Odds ratio	95% CI
**Hypothesis testing variables**						
Profession of individual completing certificate:						
Midwife	295/484	1.00	Reference	100/147	1.00	Reference
Doctor	90/165	0.68	0.45–1.04	22/32	1.02	0.44–2.37
Unknown	169/275	0.82	0.55–1.22	0/1	Insufficient variance	
No response	142/196	1.10	0.64–1.88	1/1	Insufficient variance	
Seniority of individual completing certificate:						
Junior grade	175/281	1.00	Reference	71/101	1.00	Reference
Senior grade	123/217	0.79	0.51–1.22	37/56	0.82	0.41–1.66
Unknown	218/364	0.93	0.61–1.41	14/23	0.66	0.25–1.70
No response	180/258	0.98	0.59–1.63	1/1	Insufficient variance	
**Exploratory variables: protective factors**						
Previous audit region^a^						
No	632/996	1.00	Reference	102/146	1.00	Reference
Yes	64/124	0.50	0.28–0.89	21/35	0.65	0.30–1.39
Parity:						
*Per birth >24 weeks*	*679/1094*	*0.90*	*0.82–0.99*	*123/181*	*0.76*	*0.60–0.95*
Primiparous	307/459	1.00	Reference	65/84	1.00	Reference
Multiparous	373/641	0.76	0.59–0.98	58/97	0.43	0.22–0.84
Previous stillbirth:						
*Per stillbirth*	679/1039	0.37	0.18–0.76	123/181	0.47	0.03–7.60
No	667/1070	1.00	Reference	122/179	1.00	Reference
Yes	12/28	0.42	0.19–0.94	1/2	0.47	0.03–7.60
Previous late miscarriage (18^+0^–23^+6^):						
No	652/1046	1.00	Reference	118/175	1.00	Reference
Yes	26/51	0.52	0.28–0.95	5/6	2.51	0.27–23.28
Antenatally detected congenital abnormality:						
No	551/828	1.00	Reference	26/48	1.00	Reference
Yes	143/289	0.37	0.27–0.50	97/133	0.44	0.22–0.87
Antenatal detected lethal congenital abnormality:						
No	637/988	1.00	Reference	116/166	1.00	Reference
Yes	54/125	0.27	0.18–0.42	7/15	0.38	0.13–1.10
Termination of pregnancy:						
No	627/913	1.00	Reference	110/158	1.00	Reference
Yes	69/147	0.36	0.25–0.52	13/23	0.56	0.22–1.40
Individualized birthweight centile:						
*Per five centiles*	*673/1060*	*0.97*	*0.95–0.99*	*122/180*	*0.95*	*0.90–1.00*
**Exploratory variables: adverse factors**						
Maternal body mass index (kg/m^2^):						
*Per 5 kg/m^2^*	*672/1071*	*1.18*	*1.07–1.31*	*122/178*	*1.36*	*1.07–1.73*
18.5–24.9	259/414	1.00	Reference	41/67	1.00	Reference
40+	54/67	2.73	1.34–5.53	13/13	Insufficient variance	
Individualized birthweight centile:						
<3	282/394	2.21	1.62–3.01	48/64	2.22	1.08–4.59
3–9.99	74/114	1.61	1.01–2.55	13/17	2.40	0.72–8.04
10–90	255/478	1.00	Reference	49/85	1.00	Reference
Fetal growth restriction confirmed at birth^b^:						
No	372/673	1.00	Reference	65/106	1.00	Reference
Yes	317/437	2.22	1.67–2.96	58/75	2.19	1.10–4.35
Placental weight:						
*Per 50 g*	*270/414*	*0.93*	*0.86–0.99*	*49/72*	*1.00*	*0.86–1.17*
<10^th^ centile^c^	182/256	2.12	1.33–3.37	31/43	1.58	0.58–4.31
10+ centile^c^	85/155	1.00	Reference	18/29	1.00	Reference

Summarizing the adjusted odds ratios for major error (that which is likely to alter the interpretation of the Medical Certificate of Stillbirth by family, healthcare professionals or healthcare statisticians) by hypothesis testing variables (profession and seniority) and all exploratory variables found to be associated with odds of major error within the accuracy cohort (reference group as defined in table). All odds are adjusted for clustering within hospitals through multilevel regression. For each variable/category the number of records with major error and total number of records assessed in each category are displayed along with the odds ratio, 95% CI. Due to missing data and small numbers of records in certain categories, caution should be applied when interpreting odds ratios from less frequently reported variables/categories. Further, there was insufficient power to adjust for potential confounding between different exploratory variables. To see the full results of all examined variables in the accuracy cohort and within the sensitivity cohort, please see Supplementary Table S6 (available as Supplementary data at *IJE* online).

a Hospital in previous audit region indicates a hospital that participated in the two previous audits of MCS accuracy in North-West England (Cockerill *et al.*^8^ and Higgins *et al*.^9^).

b Fetal growth restriction was defined according to the Delphi consensus definition by Gordijn *et al.*^24^ with the additional inclusion criteria of loss of >50 centiles in symphysio-fundal height in the absence of serial growth scans (provided that birthweight was below at least 50th centile).

c Gestation and sex-adjusted placental weight centiles were calculated according to Thompson *et al*.^23^

### Secondary analyses

Table 5 compares the change in primary COD ReCoDe category and key conditions between the actual and ideal MCS (frequency changes for all conditions are shown in Supplementary Table S4, available as Supplementary data at *IJE* online). Primary COD changed following adjudication in 614/1032 (59.5%; 95% CI 56.5–62.5%) cases. Six conditions were more frequently considered causative after case review: FGR, iatrogenic, placental abruption, placental insufficiency, chorioamnionitis and constricting cord knot or loop. Two documented conditions were more frequently deemed absent/non-causal: asphyxia and ‘no condition identified’. Lethal congenital abnormalities were frequently adjudicated as absent/non-causal [27/113 (23.9%; 95% CI 17.0–32.5)], particularly in terminations of pregnancy. ‘Unexplained’ stillbirth incidence reduced by almost three-quarters. FGR [195/540 (36.9%; 95% CI 32.9–41.1%)], placental insufficiency [184/540 (34.9%; 95% CI 30.9–39.0%)] and fetal (other) [49/540 (9.3%; 95% CI 7.1–12.1%)] were the most frequent reassigned causes among ‘unexplained’ cases (Supplementary Table S5, available as Supplementary data at *IJE* online).

**Table 5:** Overall primary cause of death by ReCoDe domain/condition actual Medical Certificates of Stillbirth and ideal Medical Certificates of Stillbirth after review of 1034 cases

a)		
ReCoDe Domain	Actual MCS	Ideal MCS
(A) Fetal	251 (24.3%; 21.8–27.0%)	494 (47.8%; 44.8–50.8%)
(B) Umbilical cord	7 (0.7%; 0.3–1.4%)	45 (4.4%; 3.3–5.8%)
(C) Placental	73 (7.1%; 5.7–8.8%)	238 (23.0%; 20.6–25.7%)
(D) Amniotic Fluid	17 (1.6%; 1.0–2.6%)	59 (5.7%; 4.5–7.3%)
(E) Uterine	4 (0.4%; 0.2–1.0%)	8 (0.8%; 0.4–1.5%)
(F) Maternal	39 (3.8%; 2.8–5.1%)	46 (4.5%; 3.4–5.9%)
(G) Intrapartum	46 (4.5%; 3.4–5.9%)	8 (0.8%; 0.4–1.5%)
(H) Traumatic	86 (8.3%; 6.8–10.2%)	152 (14.7%; 12.7–17.0%)
(I) Unclassified	540 (52.2%; 49.2–55.3%)	151 (14.6%; 12.6–16.9%)

**Table:** 

b)		
Condition	Actual MCS	Ideal MCS
(A7) Fetal growth restriction	49 (4.7%; 3.6–6.2%)	306 (30.0%; 26.9–32.4%)
(B2) Constricting knot or loop of cord	5 (0.5%; 0.2–1.1%)	31 (3.0%; 2.1–4.2%)
(C1) Placental abruption	58 (5.6%; 4.4–7.2%)	146 (14.1%; 12.1–16.4%)
(C4) Placental insufficiency	6 (0.6%; 0.3–1.7%)	87 (8.4%; 6.9–10.3%)
(D1) Chorioamnionitis	9 (0.9%; 0.5–1.7%)	47 (4.6%; 3.4–6.0%)
(G1) Asphyxia	46 (4.5%; 3.4–5.9%)	8 (0.8%; 0.4–1.5%)
(H2) Iatrogenic	85 (8.2%; 6.7–10.1%)	152 (14.7%; 12.7–17.0%)
(I1) No relevant condition identified	540 (52.2%; 49.2–55.3%)	150 (14.5%; 12.5–16.8%)

The primary relevant condition at death was coded according to the Relevant Condition at Death (ReCoDe) classification system (Gardosi *et al*.^22^) according to the documentation on the actual Medical Certificate of Stillbirth (MCS) issued to the parents, and according to the local data collector-constructed ideal MCS after case-note review. All cases were grouped by ReCoDe domain (a); those individual causes of death that demonstrated statistical increase or decrease after case-note review are shown in (b). The values for all individual conditions within each domain are provided in Supplementary Table S4 (available as Supplementary data at *IJE* online). Data are expressed as number (percentage of the total included cohort, *N* = 1034, for whom both actual and adjudicated primary cause of death were reported) with 95% CIs.

Overall, FGR was the most common primary COD after adjudication, present in 306/1230 (24.8%; 95% CI 22.5–27.4%) cases with an adjudicated primary COD. In 20/73 (27.4%; 95% CI 18.5–38.6%) cases originally documented as due to FGR, the FGR definition was not met. In 372 cases in which FGR was an adjudicated COD (including 66 cases in which FGR was a non-primary contributory COD), 64/372 (17.2%; 95% CI 13.7–21.4%) deaths occurred after 36* *weeks’ gestation. In these late stillbirths, 15/64 (23.4%; 95% CI 14.8–35.1%) had antenatally suspected FGR and 5 had antenatally suspected placental insufficiency. Three FGR infants born without antenatal FGR suspicion had either antenatal oligohydramnios or abnormal umbilical artery Doppler waveforms indicating suspected placental insufficiency.

Table 4 shows factors associated with major error in exploratory multilevel logistic regression (full results in Supplementary Table S6, available as Supplementary data at *IJE* online). Notable potentially protective factors included MCSs issued in previous audit region hospitals [OR 0.50 (95% CI 0.28–0.89)], previous stillbirth [OR 0.37 (95% CI 0.18–0.76) per previous loss, or OR 0.42 (95% CI 0.19–0.94) for any previous stillbirth], any previous late miscarriage [OR 0.52 (95% CI 0.28–0.95)], antenatal congenital abnormality diagnosis [any: OR 0.37 (95% CI 0.27–0.50), lethal: 0.27 (95% CI 0.18–0.42)] and pregnancy termination [OR 0.36 (95% CI 0.25–0.52)]. Notable adverse factors included: body mass index (BMI) ≥40 [OR 2.73 (95% CI 1.34–5.53)], birthweight centile <3 [OR 2.21 (95% CI 1.62–3.01)] or 3–9.9 [OR 1.61 (95% CI 1.01–2.55)], FGR confirmed after birth [OR 2.22 (95% CI 1.67–2.96)] and placental weight <10th centile [OR 2.12 (95% CI 1.33–3.37)]. Several continuous variables associated with major error odds but fell short of pre-specified OR thresholds for potential significance at examined increments (BMI, parity and birthweight centile).

### Sensitivity analysis

Full accuracy assessment was submitted in 181/1120 (16.2%) cases. Comparison between the sensitivity and remaining cohorts (data not shown) demonstrated a higher baseline antenatal care level (*P *<* *0.0001) and more frequent escalation in care during pregnancy (*P *<* *0.0001) vs cases with incomplete accuracy assessment. The error-free rate was 31/181 (17.1%; 95% CI 12.3–23.3%), minor error rate was 73/181 (40.3%; 95% CI 33.5–47.6%) and major error rate was 123/181 (68.0%; 95% CI 60.9–74.3%). Chance-adjusted accuracy was similar to the full cohort analysis: gestation Κ = 0.76 (95% CI 0.70–0.83), primary COD Κ = 0.23 (95% CI 0.17–0.31). Documented [97/181 (53.6%; 95% CI 46.3–60.7%) vs 444/883 (50.3%; 95% CI 47.0–53.6%)] and adjudicated [23/181 (12.7%; 95% CI 8.6–18.4%) vs 128/883 (14.5%; 95% CI 12.3–17.0%)] unexplained stillbirth rates were similar. FGR rates documented as causal on the original and ideal MCS were 16/181 (8.8%; 95% CI 5.5–13.9%) and 58/181 (32.0%; 95% CI 25.7–39.2%), respectively, equivalent to the remaining cohort [57/883 (6.5%; 95% CI 5.0–8.3%) and 302/883 (34.2%; 95% CI 31.1–37.4%), respectively].

The previously described major error associations with birthweight centile <3 [OR 2.22 (95% CI 1.08–4.59)], FGR [OR 2.19 (95% CI 1.10–4.35)] and congenital abnormality [OR 0.37 (95% CI 0.72–0.50)] persisted. BMI* *≥* *40 was associated with error in 13/13 (100%; 95% CI 77.5–100.0%) cases. Multiparity [OR 0.43 (95% CI 0.22–0.84)] was associated with lower odds of major error for the first time. The previously reported associations between odds of major error and previous audit region, any previous stillbirth or late miscarriage, lethal congenital abnormality, pregnancy termination, birthweight centile 3–9.9 or placental weight centile <10 were no longer demonstrated in the sensitivity cohort. The continuous variables BMI and parity, but not number of previous stillbirths or birthweight centile, all remained associated with major error (full results in Supplementary Table S6, available as Supplementary data at *IJE* online).

## Discussion

### Principal findings

MCS completion inaccuracies occurred irrespective of geographical location, with just one in five MCSs error-free. MCS issuer professional training was unrelated to major error odds. Hospitals in the previous audit region were less likely to issue a major error-containing MCS, possibly due to increased error awareness following regional initiatives.^8^^,^^9^^,^^20^ Structured assessment enables non-expert reviewers to assign likely COD in most stillbirths. FGR remains frequently undocumented on MCSs, despite being the principal COD in a quarter of cases. Placental COD (including abruption and insufficiency) were also found to be frequently underreported as cause of death after case review.

### Interpretation

Major and minor error and error-free rates, error types and chance-adjusted primary COD concordance in this geographically diverse cohort replicate prior regional UK MCS studies^8^^,^^9^ and neonatal death certificate studies.^31^ Our study reconfirms that stillbirth data accuracy is substantially worse than adult and child death certificate accuracy.^32–35^ The USA and other countries report similar issues,^36–40^ providing broader relevance for this UK study.

Case ascertainment was lower than in the MBRRACE-UK report.^12^ Given the differential characteristics of cases excluded from accuracy assessment, it is possible that excluded cases may have been more likely to have remained unexplained compared with those included in the accuracy assessment. Hospitals not participating in this study may have more errors and be less motivated to improve MCS accuracy, thus biasing our analysis in favour of inflated accuracy reporting. Similarly, the assumed accuracy of missing data (which was widespread) in this study is highly likely to be incorrect based on our sensitivity analyses. Together, our results likely overestimate true MCS accuracy nationwide. Nonetheless, MCS primary COD agreement (0.26) is unacceptable and lower than reported (between expert reviewers) in the Cause of Death and Associated Conditions perinatal classification system development (≥0.59), the only other perinatal mortality-specific classification system with reported inter-observer reliability.^41^ Although there is controversy regarding the ‘best’ perinatal death classification system, ReCoDe was chosen because it includes FGR and placental causes (leading UK preventable stillbirth causes and essential criteria of global stillbirth classification systems) as direct CODs.^42^

COD assessment guidelines for stillbirth may improve accuracy. Certificates issued from the previous audit region, where regional guidelines for structured case review and MCS completion are provided,^20^ demonstrated fewer major errors. Specific birthweight centile and growth trajectory assessment increased FGR identification, which may not be obvious from birthweight. Many stillborn FGR infants in this study had exceeded recommended gestation-related delivery thresholds^5^ and had excellent neonatal survival chances.^43^ Some such FGR deaths might have been preventable; this should be continually audited.

MCS issuer professional training appears unrelated to the major error likelihood, mirroring the pattern in adult death certification.^44^ It is notable that some factors protective against major MCS error are those associated with increased risk of stillbirth such as previous stillbirth or congenital abnormalities; this may reflect increased awareness and contemporaneous documentation of factors predisposing to stillbirth in such women.^11^ The persistent association of birthweight <3rd centile and FGR diagnosis (confirmed post-birth) with increased major error odds likely reflects specific FGR diagnosis assignment instruction provision to local data collectors, whereas original MCS issuers had no such instruction. Similarly, local definitions for other conditions may have introduced variation in assignment across the cohort. However, assignment frequency in many conditions changed after case review.

### Strengths

We have demonstrated that using a structured case review, non-expert reviewers are able to assign a COD for the majority of stillbirths without specific training. Utilizing a similar method and the same perinatal death classification system as used in previous North-West England studies allows comparisons over time; this study provides greater accuracy assessment depth compared with previous studies by examining secondary COD and related condition data. Kappa statistical analysis allows discernment between agreement by chance and systematic agreement.

Over-attribution to FGR has been minimized using a restricted definition, birthweight centile calculations that adjust for post-mortem *in utero* retention and considering all conditions’ relevant contributions, rather than adhering to the ReCoDe hierarchical structure. This allowed alternative primary COD assignment (e.g. placental abruption) where appropriate, even in FGR infants.^45^ Use of the stillborn FGR definition would have been ideal, but this was published after data collection commenced^46^ and relies on autopsy results, which were unavailable contemporaneously as explained above. Restricting case-note review to contemporaneous data ensures the adjudicated COD could have been contemporaneously assigned. Unless stillbirth certification/registration systems are redesigned to allow amendment for delayed post-mortem investigation, as in Wisconsin (USA),^47^ individual and nationally reported stillbirth CODs will remain dependent on the individual certifier and case-review quality.

### Limitations

Low sample size with high missing data rates impaired detection of associations between patient/healthcare variables and major error odds, and prevented multivariable adjustment for potential confounding. Future studies should be appropriately powered to confirm or refute the associations we found. Caution should particularly be applied when interpreting sensitivity analysis data (83% cases excluded), which was underpowered to detect/confirm potential clinically important relationships. Despite this, the results remain meaningful and informative. Although included case characteristics (birthweight, gestation, maternal ethnicity) are similar to those in national reports,^12^^,^^28–30^ it is not possible to compare the reported and unreported case characteristics as patient identity protection outside the direct clinical care team was paramount. Finally, distinction between major and minor errors may be semantic. However, those errors defined as ‘minor’ would not independently result in management change or alter retrospective evaluation of antemortem management and therefore deserve less attention.

## Conclusion

We identified nationwide stillbirth certification and COD assignment deficiencies, confirming our first hypothesis. This reinforces the need for independent, national perinatal surveillance processes such as MBRRACE-UK. However, the lack of evidence that MCS accuracy relates to issuer profession and seniority, combined with the lower odds of major error in previous audit region hospitals, suggests that basic tools, guidance and awareness raising among individual MCS certifiers and hospitals may greatly help to improve data quality. Correct stillbirth cause classification is crucial for families and society; when ‘unexplained’, conditions’ true perinatal mortality contributions are uncounted and preventative strategies cannot be appropriately targeted. Without this, the UK and other countries will fail to meet international stillbirth reduction targets. Therefore, it is imperative to ensure MCS accuracy and to undertake a continuous feedback and performance improvement cycle. Until stillbirth certification systems allow COD amendments, individual and nationally reported stillbirth CODs will remain dependent on the individual certifier and case-review quality.

## Notes

The UKARCOG NESTT working group: Aadan H, Ajoku KF, Asim S, Badger EJ, Barfi LE, Bevington LM, Bhat M, Black N, Black RE, Boughey SA, Brewster CB, Buchanan CE, Bullough SH, Byrne V, Carpenter CC, Carron SA, Conti-Ramsden F, Cordell VC, Craggs S, Creswell LC, Cury Fernandes A, Dalton JA, Dracocardos DV, Dunlop CE, Egan JK, Ejiofor CI, Elderfield CHJ, Faluyi D, Geddes-Barton DM, Giacchino T, Giles SE, Goodier EA, Goodman JK, Govil M, Grainger R, Guyett EA, Haken AJ, Harrison RC, Hartley JL, Hogg FS, Hutchinson E, Jamie HS, Jamison LAC, Jaufuraully S, Jethwani G, Jones CM, Karapanos I, Karavadra B, Kasaven LS, Kaur R, Kermack AJ, King A, Lallemant CLB, Lattey KR, Layden EA, MacMahon CA, Maddy LR, Magee SM, Mahilchi Sudar L, Mahmud A, Marks KJ, McNally A, Medford ES, Milliken SVI, Mogekwu OM, Mohamed HE, Moorhouse SS, Mouhajer M, Mumtaz H, Murphy LL, Navaratnam K, Neville AE, Nijjar SK, O’Brien S, Obeysekera MH, Odonde RI, Ofodile O, Okagbue N, Parnell L, Pearce MD, Petrovic M, Picart SR, Plant CL, Powell HM, Powell SE, Preston L, Raglan O, Ramcharn M, Reilly KM, Riaz N, Riches JM, Rutherford DR, Sathyendran P, Shamsudin F, Simonian M, Smith KE, Sohal-Burnside JK, Standing LJ, Stirrat LI, Stocker LJ, Subba K, Summerhill C, Taylor CJ, Thomson S, Thyne KCA, Tomlinson F, Troko J, Verasingam N, Welch HE, Whittle KM, Williams SG, Wilson KJ, Wilson SF, Wilson-Theaker WP, Wyeth CLM.

## Ethics approval

According to the HRA decision tool (http://www.hra-decisiontools.org.uk/ethics/), approvals were not required for this service evaluation in which no patient-identifying data or personal data were accessed by individuals outside the direct healthcare team.

## Supplementary Material

dyac100_Supplementary_Data

## Data Availability

The data underlying this article are available in the Figshare data repository, University of Manchester, at https://dx.doi.org/10.48420/14330090.

## References

[1] Still-Birth (Definition) Act 1992, Chapter 29. https://www.legislation.gov.uk/ukpga/1992/29/section/1 (11 May 2022, date last accessed).

[2] Office for National Statistics. Child and Infant Mortality in England and Wales: 2018. London: The Stationery Office, 2020.

[3] Stillbirth and Neonatal Death Society. What happens at a hospital post-mortem on a baby —procedures and likely timings. https://www.sands.org.uk/sites/default/files/What%20happens%20at%20a%20post%20mortem%20-%20procedures%20and%20timings.pdf (3 February 2022, date last accessed).

[4] Office for National Statistics. Child and Infant Mortality Statistics QMI. London: The Stationery Office, 2021.

[5] NHS England. Saving Babies’ Lives Care Bundle Version 2. London: The Stationery Office, 2019.

[6] Office for National Statistics and HM Passport Office. Guidance for Doctors Completing Medical Certificates of Cause of Death in England and Wales. London: The Stationery Office, 2020.

[7] Coroners and Justice Act (c.1). London: The Stationery Office, 2009.

[8] Cockerill R , Whitworth MK, Heazell AEP. Do medical certificates of stillbirth provide accurate and useful information regarding the cause of death? Paediatr Perinat Epidemiol 2012;26:117–23. 22324497 10.1111/j.1365-3016.2011.01247.x

[9] Higgins LE , Heazell AEP, Whitworth MK. Persistent inaccuracies in completion of medical certificates of stillbirth: a cross-sectional study. Paediatr Perinat Epidemiol 2018;32:474–81. 30300448 10.1111/ppe.12501 PMC6221058

[10] Pritt BS , Hardin NJ, Richmond JA, Shapiro S. Death certification errors at an academic institution. Arch Pathol Lab Med 2005;129:1476–79. 16253030 10.5858/2005-129-1476-DCEAAA

[11] Sissakos D , Fox R, Draycott T, Winter C. Late Intrauterine Fetal Death and Stillbirth. Green-top Guideline No. 55. London: Royal College of Obstetricians and Gynaecologists, 2010.

[12] Draper ES , Gallimore ID, Smith LK et al MBRRACE-UK Perinatal Mortality Surveillance Report: UK Perinatal Deaths for Births from January to December 2018. Leicester: The Infant Mortality and Morbidity Studies Department of Health Sciences, University of Leicester, 2020.

[13] Stevens JD , Landes SD. Assessing state level variation in signature authority and cause of death accuracy, 2005-2017. Prev Med Rep 2021;21:101309. 33511026 10.1016/j.pmedr.2020.101309 PMC7815989

[14] Johnson CJ , Hahn CG, Fink AK, German RR. Variability in cancer death certificate accuracy by characteristics of death certifiers. Am J Forensic Med Pathol 2012;33:137–42. 21490500 10.1097/PAF.0b013e318219877e PMC12305805

[15] UNICEF and World Health Organisation. Ending preventable newborn deaths and stillbirths by 2030. https://data.unicef.org/resources/ending-preventable-newborn-deaths-and-stillbirths-by-2030/ (5 February 2022, date last accessed).

[16] United Kingdom Audit and Research Collaborative in Obstetrics and Gynaecology. About Us. http://ukarcog.org/about-us/ (31 January 2021, date last accessed).

[17] Health Education England. How we work: in your area. https://www.hee.nhs.uk/about/how-we-work/your-area (22 February 2022, date last accessed).

[18] Royal College of Obstetricians and Gynaecologists. Registration of Stillbirths and Certification for Pregnancy Loss before 24 Weeks of Gestation (Good Practice No. 4). London: Royal College of Obstetricians and Gynaecologists, 2005. 15984641

[19] Al Wattar BH , Tamblyn JA, Parry-Smith W, Prior M, Van Der Nelson H. Management of obstetric postpartum hemorrhage: a national service evaluation of current practice in the UK. RMHP 2017; 10:1–6. 10.2147/RMHP.S121737 PMC5261839 28176919

[20] Health Innovation Manchester. Management of Stillbirth Integrated Care Pathway. https://healthinnovationmanchester.com/wp-content/uploads/2018/10/NW-Stillbirth-ICP-V3-March-2018.pdf (22 February 2022, date last accessed).

[21] NHS England. 2013/2014 NHS standard contract for perinatal pathology: Particulars, Schedule 2—the services, A—service specification. https://www.england.nhs.uk/wp-content/uploads/2013/06/e12-perinatal-path.pdf (8 February 2022, date last accessed).

[22] Gardosi J , Kady SM, McGeown P, Francis A, Tonks A. Classification of stillbirth by Relevant Condition at Death (ReCoDe): population based cohort study. BMJ 2005;331:1113–17. 16236774 10.1136/bmj.38629.587639.7C PMC1283273

[23] Thompson JMD , Irgens LM, Skjaerven R, Rasmussen S. Placental weight percentile curves for singleton deliveries. BJOG 2007;114:715–20. 17516963 10.1111/j.1471-0528.2007.01327.x

[24] Gordijn SJ , Beune IM, Thilaganathan B et al Consensus definition of fetal growth restriction: a Delphi procedure. Ultrasound Obstet Gynecol 2016;48:333–39. 26909664 10.1002/uog.15884

[25] Chitty LS , Altman DG, Henderson A, Campbell S. Charts of fetal size: 3. Abdominal measurements. Br J Obstet Gynaecol 1994;101:125–31. 8305386 10.1111/j.1471-0528.1994.tb13077.x

[26] Heazell A , Li M, Budd J et al Association between maternal sleep practices and late stillbirth: findings from a stillbirth case-control study. BJOG 2018;125:254–62. 29152887 10.1111/1471-0528.14967 PMC5765411

[27] Landis JR , Koch GG. The measurement of observer agreement for categorical data. Biometrics 1977;33:159–74. 843571

[28] Office for National Statistics. Birth Characteristics: 2018. London: The Stationery Office, 2019

[29] National Records of Scotland. Vital Events Reference Tables 2018. Edinburgh: National Records of Scotland Web Archive, 2020.

[30] Northern Ireland Statistics and Research Agency. Registrar General Annual Report 2018: Stillbirths and Infant Deaths. Belfast: Northern Ireland Statistics and Research Agency, 2019.

[31] Hunt R , Barr P. Errors in the certification of neonatal death. J Paediatr Child Health 2000;36:498–501. 11036809 10.1046/j.1440-1754.2000.00556.x

[32] Krywanczyk A , Amoresano E, Tatsumi K, Mount S. Autopsy service death certificate review. Arch Pathol Lab Med 2020;144:1092–96. 31986077 10.5858/arpa.2019-0452-OA

[33] Wood KA , Weinberg SH, Weinberg ML. Death certification in Northern Alberta: error occurrence rate and educational intervention. Am J Forensic Med Pathol 2020;41:11–17. 31977347 10.1097/PAF.0000000000000527

[34] Miki J , Rampatige R, Richards N, Adair T, Cortez-Escalante J, Vargas-Herrera J. Saving lives through certifying deaths: assessing the impact of two interventions to improve cause of death data in Peru. BMC Public Health 2018;18:1329. 30509233 10.1186/s12889-018-6264-1 PMC6276144

[35] Gupta N , Bharti B, Singhi S, Kumar P, Thakur JS. Errors in filling WHO death certificate in children: lessons from 1251 death certificates. J Trop Pediatr 2014;60:74–78. 23902671 10.1093/tropej/fmt059

[36] de Almeida MF , Alencar GP, Schoeps D. Quality of information registered on fetal deaths certificates in Sao Paulo, Southeastern Brazil. Rev Saude Publica 2011;45:845–53. 21845290 10.1590/s0034-89102011005000058

[37] Lee EJ , Gambatese M, Begier E, Soto A, Das T, Madsen A. Understanding perinatal death: a systematic analysis of New York City fetal and neonatal death vital record data and implications for improvement, 2007-2011. Matern Child Health J 2014;18:1945–54. 24522520 10.1007/s10995-014-1440-0

[38] dos Santos Barbeiro FM , Costa Fonseca S, Tauffer MG et al Fetal deaths in Brazil: a systematic review. Rev Saude Publica 2015;49:22. 25902565 10.1590/S0034-8910.2015049005568 PMC4390075

[39] Harrist AV , Busacker A, Kroelinger CD. Evaluation of the completeness, data quality, and timeliness of fetal mortality surveillance in Wyoming, 2006-2013. Matern Child Health J 2017;21:1808–13. 28744700 10.1007/s10995-017-2323-y

[40] Christiansen-Lindquist L , Silver RM, Parker CB et al Fetal death certificate data quality: a tale of two US counties. Ann Epidemiol 2017;27:466–71.e2. 28789821 10.1016/j.annepidem.2017.07.001 PMC5610070

[41] Froen JF , Pinar H, Flenady V et al Causes of death and associated conditions (Codac): a utilitarian approach to the classification of perinatal deaths. BMC Pregnancy Childbirth 2009;9:22. 19515228 10.1186/1471-2393-9-22 PMC2706222

[42] Wojcieszek AM , Reinebrant HE, Hopkins Leisher S et al Characteristics of a global classification system for perinatal deaths: a Delphi consensus study. BMC Pregnancy Childbirth 2016;16:223. 27527704 10.1186/s12884-016-0993-x PMC4986199

[43] Santhakumaran S , Statnikov Y, Gray D et al Survival of very preterm infants admitted to neonatal care in England 2008-2014: time trends and regional variation. Arch Dis Child Fetal Neonatal Ed 2018;103:F208–15. 28883097 10.1136/archdischild-2017-312748 PMC5916099

[44] James DS , Bull AD. Death certification: is correct formulation of cause of death related to seniority or experience? J R Coll Physicians Lond 1995;29:424–28. 8847688 PMC5401209

[45] Ego A , Zeitlin J, Batailler P et al Stillbirth classification in population-based data and role of fetal growth restriction: the example of ReCoDe. BMC Pregnancy Childbirth 2013;13:182. 24090495 10.1186/1471-2393-13-182 PMC3850812

[46] Beune IM , Damhuis SE, Ganzevoort W et al Consensus definition of fetal growth restriction in intrauterine fetal death: a Delphi procedure. Arch Pathol Lab Med 2021;145:428–36. 32882006 10.5858/arpa.2020-0027-OA

[47] Brooks EG , Reed KD. Principles and pitfalls: a guide to death certification. Clin Med Res 2015;13:74–82. 26185270 10.3121/cmr.2015.1276 PMC4504663

