# Supplementary material for: Worth the paper it’s written on? A cross-sectional study of Medical Certificate of Stillbirth accuracy in the UK
Source: Int J Epidemiol. 2022 Jun 20;52(1):295–308. doi: 10.1093/ije/dyac100 (PMC9908049; doi:10.1093/ije/dyac100)
Supplement: dyac100_Supplementary_Data [file dyac100_supplementary_data.zip › dyac100_Supplementary_Data/ije-2021-09-1359-File006.docx]

Supplementary data

**Supplementary Table S1: STROBE Statement Checklist**

|  | **Item No** | **Recommendation** | **Page**  **No** |
| --- | --- | --- | --- |
| **Title and abstract** | 1 | (*a*) Indicate the study’s design with a commonly used term in the title or the abstract | 1 |
|  |  | (*b*) Provide in the abstract an informative and balanced summary of what was done and what was found | 2 |
| **Introduction** | | | |
| Background/rationale | 2 | Explain the scientific background and rationale for the investigation being reported | 4-5 |
| Objectives | 3 | State specific objectives, including any prespecified hypotheses | 5 |
| **Methods** | | | |
| Study design | 4 | Present key elements of study design early in the paper | 6 |
| Setting | 5 | Describe the setting, locations, and relevant dates, including periods of recruitment, exposure, follow-up, and data collection | 6  Table S3 |
| Participants | 6 | (*a*) Give the eligibility criteria, and the sources and methods of selection of participants | 6 |
| Variables | 7 | Clearly define all outcomes, exposures, predictors, potential confounders, and effect modifiers. Give diagnostic criteria, if applicable | 7-8  Table S3  Table S6 |
| Data sources/ measurement | 8* | For each variable of interest, give sources of data and details of methods of assessment (measurement). Describe comparability of assessment methods if there is more than one group | 6-8 |
| Bias | 9 | Describe any efforts to address potential sources of bias | 7 |
| Study size | 10 | Explain how the study size was arrived at | 7 |
| Quantitative variables | 11 | Explain how quantitative variables were handled in the analyses. If applicable, describe which groupings were chosen and why | 10 |
| Statistical methods | 12 | (*a*) Describe all statistical methods, including those used to control for confounding | 10 |
|  |  | (*b*) Describe any methods used to examine subgroups and interactions | 10 |
|  |  | (*c*) Explain how missing data were addressed | 9 |
|  |  | (*d*) If applicable, describe analytical methods taking account of sampling strategy | N/A |
|  |  | (*e*) Describe any sensitivity analyses | 10 |
| **Results** | | | |
| Participants | 13* | (a) Report numbers of individuals at each stage of study—eg numbers potentially eligible, examined for eligibility, confirmed eligible, included in the study, completing follow-up, and analysed | 11  Tables 3-5  All suppl tables  Fig 2 |
|  |  | (b) Give reasons for non-participation at each stage | 11  Fig. 2 |
|  |  | (c) Consider use of a flow diagram | Fig. 2 |
| Descriptive data | 14* | (a) Give characteristics of study participants (eg demographic, clinical, social) and information on exposures and potential confounders | 11  Table S3 |
|  |  | (b) Indicate number of participants with missing data for each variable of interest | Tables 3-5  All suppl. tables  Figure 2 |
| Outcome data | 15* | Report numbers of outcome events or summary measures | Tables 3-5  All suppl. tables  Figures 2-3 |
| Main results | 16 | (*a*) Give unadjusted estimates and, if applicable, confounder-adjusted estimates and their precision (eg, 95% confidence interval). Make clear which confounders were adjusted for and why they were included | 11-14  Tables 3-5  All suppl. Tables  Figure 3 |
|  |  | (*b*) Report category boundaries when continuous variables were categorized | N/A |
|  |  | (*c*) If relevant, consider translating estimates of relative risk into absolute risk for a meaningful time period | N/A |
| Other analyses | 17 | Report other analyses done—eg analyses of subgroups and interactions, and sensitivity analyses | 14 |
| **Discussion** | | | |
| Key results | 18 | Summarise key results with reference to study objectives | 15 |
| Limitations | 19 | Discuss limitations of the study, taking into account sources of potential bias or imprecision. Discuss both direction and magnitude of any potential bias | 18 |
| Interpretation | 20 | Give a cautious overall interpretation of results considering objectives, limitations, multiplicity of analyses, results from similar studies, and other relevant evidence | 15-17 |
| Generalisability | 21 | Discuss the generalisability (external validity) of the study results | 15 |
| **Other information** | | | |
| Funding | 22 | Give the source of funding and the role of the funders for the present study and, if applicable, for the original study on which the present article is based | 21 |

Page numbers refer to the submitted manuscript document.

**Supplementary File S2 - data collection tool**

The local data collection tool is provided in Excel format (Supplementary File S2 – data collection tool.xlsx) for others to use to extract and audit comparable data from retrospective maternal records and Medical Certificates of Stillbirth. Care should be taken to anonymise any data collected prior to sharing outside of the direct care team.

**Supplementary Table S3: Characteristics of included pregnancies, including comparison of cases included and excluded from accuracy assessment (n=1,246)**

|  | All  (N=1,246) | Accuracy assessed  (N=1,120) | Accuracy not assessed  (N=126) | | Assessed vs. Not assessed  P |
| --- | --- | --- | --- | --- | --- |
| **Healthcare variables** | | | | | |
| Country | | | | | |
| England | 946/1,246 (75.9%; 73.5-78.2%) | 862/1,120 (77.0%; 74.4-79.3%) | 84/126 (66.7%; 58.1-74.3) | | <0.0001 |
| Wales | 89/1,246 (7.1%; 5.8-8.7%) | 80/1,120 (7.1%; 5.8-8.8%) | 9/126 (7.1%; 3.8-13.0%) | |  |
| Scotland | 147/1,246 (11.8%; 10.1-13.7%) | 115/1,120 (10.3%; 8.6-12.2%) | 32/126 (25.4%; 18.6-33.7%) | |  |
| N. Ireland | 64/1,246 (5.1%; 4.1-6.5%) | 63/1,120 (5.6%; 4.4-7.1%) | 1/126 (0.8%; 0.1-4.4%) | |  |
| Previous audit region | | | | | |
| Yes | 139/1,246 (11.2%; 9.5-13.0%) | 124/1,120 (11.1%; 9.4-13.0%) | 15/126 (11.9%; 7.3-18.7%) | 0.78 | |
| No | 1,107/1,246 (88.8%; 87.0-90.5%) | 996/1,120 (88.9%; 87.0-90.6%) | 111/126 (88.1%; 81.3-92.7%) |  |  |
| Maternity Care | | | | | |
| Secondary | 659/1,246 (52.9%; 50.1-55.7%) | 581/1,120 (51.9%; 49.0-54.8%) | 78/126 (61.9%; 53.2-70.0%) | | 0.032 |
| Tertiary | 587/1,246 (47.1%; 44.4-49.9%) | 539/1,120 (48.1%; 45.2-51.1%) | 48/126 (38.1%; 30.1-46.8%) | |  |
| Neonatal Care | | | | | |
| Level 1 | 8/1,246 (0.6%; 0.3-1.3%) | 8/1,120 (0.7%; 0.4-1.4%) | 0/126 (0.0%; 0.0-3.0%) | | 0.33 |
| Level 2 | 511/1,246 (41.0%; 38.3-43.8%) | 453/1,120 (40.5%; 37.6-43.4%) | 58/126 (46.0%; 37.6-54.7%) | |  |
| Level 3 | 727/1,246 (58.4%; 55.6-61.1%) | 659/1,120 (58.8%; 55.9-61.7) | 68/126 (54.0%; 45.3-62.4%) | |  |
| **Maternal / Baseline variables** | | | | | |
| *Maternal age (years)* | *31 (26-35)* | *31 (26-35)* | *31 (27-34)* | | *0.43* |
| Age >35 | 323/1,231 (26.2%; 23.9-28.8%) | 295/1,110 (26.6%; 24.1-29.3%) | 28/121 (23.1%; 16.5-31.4%) | | 0.42 |
| *Maternal body mass index (BMI) (kg/m^2^)* | *26.3 (22.8-31.5)* | *26.4 (22.7-31.5)* | *27.1 (23.4-31.4)* | | *0.48* |
| BMI >30 | 372/1,195 (31.1%; 28.6-33.8%) | 333/1,071 (31.1%; 28.4-33.9%) | 39/124 (31.5%; 23.9-40.0%) | | 0.94 |
| Ethnic category | | | | | |
| White | 888/1,237 (71.8%; 69.2-74.2%) | 801/1,112 (72.0%; 69.3-74.6%) | 87/125 (69.6%; 61.1-77.0%) | | 0.93 |
| Black | 102/1,237 (8.3%; 6.8-9.9%) | 90/1,112 (8.1%; 6.6-9.8%) | 12/125 (9.6%; 5.6-16.0%) | |  |
| Asian | 152/1,237 (12.3%; 10.6-14.2%) | 136/1,112 (12.2%; 10.4-14.3%) | 16/125 (12.8%; 8.0-19.8%) | |  |
| Other (not stated) | 95/1,237 (7.7%; 6.3-9.3%) | 85/1,112 (7.6%; 6.2-9.4%) | 10/125 (8.0%; 4.4-14.1%) | |  |
| Smoker | 236/1,204 (19.6%; 17.5-21.9%) | 215/1,079 (19.9%; 17.7-22.4%) | 21/125 (16.8%; 11.3-24.3%) | | 0.41 |
| Alcohol | 19/1,187 (1.6%; 1.0-2.5%) | 18/1,064 (1.7%; 1.1-2.7%) | 1/123 (0.8%; 0.1-4.5%) | | 0.46 |
| Recreational drug abuse | 30/1,172 (2.6%; 1.8-3.6%) | 28/1,047 (2.7%; 1.9-3.8%) | 2/125 (1.6%; 0.4-5.7%) | | 0.47 |
| *Parity* | *1 (0-2)* | *1 (0-2)* | *1 (0-2)* | | *0.80* |
| Primiparous | 511/1,223 (41.8%; 39.1-44.6%) | 459/1,100 (41.7%; 38.9-44.7%) | 52/123 (42.3%; 33.9-51.1%) | | 0.91 |
| Previous stillbirth | 32/1,221 (2.6%; 1.9-3.7%) | 28/1,098 (2.6%; 1.8-3.7%) | 4/123 (3.3%; 1.3-8.1%) | | 0.64 |
| Previous pregnancy loss 18-23^+6^ weeks | 57/1,220 (4.7%; 3.6-6.0%) | 51/1,097 (4.7%; 3.6-6.1%) | 6/123 (4.9%; 2.3-10.2%) | | 0.91 |
| **Pregnancy / Antenatal variables** | | | | | |
| Number of fetuses | | | | | |
| Singleton | 1,171/1,244 (94.1%; 92.7-95.3%) | 1,051/1,118 (94.0%; 92.5-95.3%) | 120/126 (95.2%; 90.0-97.8%) | | 0.73 |
| Twin | 69/1,244 (5.6%; 4.4-7.0%) | 63/1,118 (5.6%; 4.4-7.2%) | 6/126 (4.8%; 2.2-10.0%) | |  |
| Triplet | 4/1,244 (0.3%; 0.1-0.8%) | 4/1,118 (0.4%; 0.1-0.9%) | 0/126 (0.0%; 0.0-3.0%) | |  |
| *Gestation at first contact (days)* | *72 (60-89)* | *71 (59-89)* | *74 (61-91)* | | *0.28* |
| Booking after 18 weeks’ gestation | 140/1,186 (11.8%; 10.1-13.8%) | 125/1,066 (11.7%; 9.9-13.8%) | 15/120 (12.5%; 7.7-19.6%) | | 0.80 |
| Unbooked pregnancy | 1/1,233 (0.1%; 0.0-0.5%) | 1/1,109 (0.1%; 0.0-0.5%) | 0/124 (0.0%; 0.0-3.0%) | | 0.74 |
| Initial level of care | | | | | |
| None | 1/1,233 (0.1%; 0.0-0.5%) | 1/1,109 (0.1%; 0.0-0.5%) | 0/124 (0.0%; 0.0-3.0%) | | 0.011 |
| Midwife-led | 503/1,233 (40.8%; 38.1-43.6%) | 444/1,109 (40.0%; 37.2-43.0%) | 59/124 (47.6%; 39.0-56.3%) | |  |
| Consultant-led | 613/1,233 (49.7%; 46.9-52.5%) | 567/1,109 (51.1%; 48.2-54.1%) | 46/124 (37.1%; 29.1-45.9%) | |  |
| Specialist-led | 116/1,233 (9.4%; 7.9-11.2%) | 97/1,109 (8.8%; 7.2-10.6%) | 19/124 (15.3%; 10.0-22.7%) | |  |
| Highest level of care | | | | | |
| None | 1/1,236 (0.1%; 0.0-0.5%) | 1/1,113 (0.1%; 0.0-0.5%) | 0/123 (0.0%; 0.0-3.0%) | | 0.93 |
| Midwife-led | 72/1,236 (5.8%; 4.7-7.3%) | 64/1,113 (5.8%; 4.5-7.3%) | 8/123 (6.5%; 3.3-12.3%) | |  |
| Consultant-led | 654/1,236 (52.9%; 50.1-55.7%) | 587/1,113 (52.7%; 49.8-55.7%) | 67/123 (54.5%; 45.7-63.0%) | |  |
| Specialist-led | 509/1,236 (41.2%; 38.5-44.0%) | 461/1,113 (41.4%; 38.6-44.3%) | 48/123 (39.0%; 30.9-47.9%) | |  |
| Escalation of care in pregnancy | 615/1,232 (49.9%; 47.1-52.7%) | 548/1,109 (49.4%; 46.5-52.4%) | 67/123 (54.5%; 45.7-63.0%) | | 0.29 |
| Antenatally detected congenital abnormality | 314/1,243 (25.3%; 22.9-27.8%) | 289/1,117 (25.9%; 23.4-28.5%) | 25/126 (19.8%; 13.8-27.7%) | | 0.14 |
| Lethal abnormality | 132/1,239 (10.7%; 9.1-12.5%) | 125/1,113 (11.2%; 9.5-13.2%) | 7/126 (5.6%; 2.7-11.0%) | | 0.050 |
| Ultrasound fetal growth assessment(s) | 686/1,244 (55.1%; 52.4-57.9%) | 617/1,118 (55.2%; 52.3-58.1%) | 69/126 (54.8%; 46.1-63.2%) | | 0.93 |
| *Number of scans* | *1 (0-2)* | *1 (0-2)* | *1 (0-2)* | | *0.92* |
| Oligohydramnios | 110/1,106 (10.0%; 8.3-11.9%) | 100/1,010 (9.9%; 8.2-11.9%) | 10/96 (10.4%; 5.8-18.1%) | | 0.87 |
| Abnormal umbilical artery Doppler | 129/993 (13.0%; 11.0-15.2%) | 120/914 (13.1%; 11.1-15.5%) | 9/79 (11.4%; 6.1-20.3%) | | 0.66 |
| Abnormal other Doppler | 81/938 (8.6%; 7.0-10.6%) | 76/875 (8.7%; 7.0-10.7%) | 5/63 (7.9%; 3.4-17.3%) | | 0.84 |
| Antenatal fetal growth assessment | | | | | |
| Normal/no concern | 527/1,021 (51.6%; 48.6-54.7%) | 464/928 (50.0%; 46.8-53.2%) | 63/93 (67.7%; 57.7-76.4%) | | 0.014 |
| SGA/FGR/static | 257/1,021 (25.2%; 22.6-27.9%) | 241/928 (26.0%; 23.3-28.9%) | 16/93 (17.2%; 10.9-26.1%) | |  |
| LGA/accelerative | 64/1,021 (6.3%; 4.9-7.9%) | 60/928 (6.5%; 5.1-8.2%) | 4/93 (4.3%; 1.7-10.5%) | |  |
| Too early to assess | 173/1,021 (16.9%; 14.8-19.4%) | 163/928 (17.6%; 15.3-20.1%) | 10/93 (10.8%; 5.9-18.7%) | |  |
| Termination of pregnancy | 164/1,241 (13.2%; 11.5-15.2%) | 151/1,120 (13.5%; 11.6-15.6%) | 13/121 (10.7%; 6.4-17.5%) | | 0.40 |
| **Diagnosis / Birth variables** | | | | | |
| Sex | | | | | |
| Male | 629/1,237 (50.9%; 48.1-53.6%) | 566/1,116 (50.7%; 47.8-53.7%) | 63/121 (52.1%; 43.2-60.8%) | | 0.44 |
| Female | 593/1,237 (47.9%; 45.2-50.7%) | 535/1,116 (47.9%; 45.0-50.9%) | 58/121 (47.9%; 39.2-56.8%) | |  |
| Unable to identify | 15/1,237 (1.2%; 0.7-2.0%) | 15/1,116 (1.3%; 0.8-2.2%) | 0/121 (0.0%; 0.0-3.1%) | |  |
| *Gestation at diagnosis of fetal death in utero (weeks^+days^)* | *32^+0^ (26^+6^-37^+0^)* | *31^+5^ (26^+5^-36^+5^)* | *33^+3^ (27^+0^-38^+2^)* | | *0.064* |
| Gestation <24^+0 a^ | 24/1,209 (2.0%; 1.3-2.9%) | 23/1,090 (2.1%; 1.4-3.2%) | 1/119 (0.8%; 0.2-4.6%) | | 0.24 |
| Gestation 24^+0^-27^+6^ | 357/1,209 (29.5%; 27.0-32.2%) | 322/1,090 (29.5%; 26.9-32.3%) | 35/119 (29.4%; 22.0-38.1%) | |  |
| Gestation 28^+0^-33^+6^ | 315/1,209 (26.1%; 23.7-28.6%) | 290/1,090 (26.6%; 24.1-29.3%) | 25/119 (21.0%; 14.7-29.2%) | |  |
| Gestation 34^+0^-36^+6^ | 210/1,209 (17.4%; 15.3-19.6%) | 192/1,090 (17.6%; 15.5-20.0%) | 18/119 (15.1%; 9.8-22.7%) | |  |
| Gestation 37^+0^-40^+6^ | 272/1,209 (22.5%; 20.2-24.9%) | 237/1,090 (21.7%; 19.4-24.3%) | 35/119 (29.4%; 22.0-38.1%) | |  |
| Gestation ≥41^+0^ | 31/1,209 (2.6%; 1.8-3.6%) | 26/1,090 (2.4%; 1.6-3.5%) | 5/119 (4.2%; 1.8-9.5%) | |  |
| Timing of diagnosis of fetal death *in utero* | | | | | |
| Antenatal | 1,104/1,231 (89.7%; 87.9-91.3%) | 997/1,111 (89.7%; 87.8-91.4%) | 107/120 (89.2%; 82.4-93.6%) | | 0.62 |
| Intrapartum | 100/1,231 (8.1%; 6.7-9.8%) | 88/1,111 (7.9%; 6.5-9.7%) | 12/120 (10.0%; 5.8-16.7%) | |  |
| Postpartum | 5/1,231 (0.4%; 0.2-1.0%) | 5/1,111 (0.5%; 0.2-1.1%) | 0/120 (0.0%; 0.0-3.1%) | |  |
| Unknown | 22/1,231 (1.8%; 1.2-2.7%) | 21/1,111 (1.9%; 1.2-2.9%) | 1/120 (0.8%; 0.2-4.6%) | |  |
| *Diagnosis to delivery interval (days)* | *2 (0-3)* | *2 (0-3)* | *1 (0-2)* | | *0.0060* |
| Interval > 1 week | 27/1,215 (2.2%; 1.5-3.2%) | 26/1,098 (2.4%; 1.6-3.5%) | 1/117 (0.9%; 0.2-4.7%) | | 0.31 |
| *Birthweight (grams)* | *1520 (740-2580)* | *1512 (735-2560)* | *1800 (760-2880)* | | *0.14* |
| Birthweight <1500g | 608/1,241 (49.0%; 46.2-51.8%) | 554/1,118 (49.6%; 46.6-52.5%) | 54/123 (43.9%; 35.5-52.7%) | | 0.34 |
| Birthweight 1500-2499.9g | 296/1,241 (23.9%; 21.6-26.3%) | 269/1,118 (24.1%; 21.7-26.7%) | 27/123 (22.0%; 15.6-30.1%) | |  |
| Birthweight 2500-4000g | 320/1,241 (25.8%; 23.4-28.3%) | 280/1,118 (25.0%; 22.6-27.7%) | 40/123 (32.5%; 24.9-41.2%) | |  |
| Birthweight >4000g | 17/1,241 (1.4%; 0.9-2.2%) | 15/1,118 (1.3%; 0.8-2.2%) | 2/123 (1.6%; 0.5-5.7%) | |  |
| *Individualised birthweight centile* | *15.0 (0.5-50.7)* | *13.9 (0.5-51.8)* | *18.9 (0.6-47.1)* | | *0.52* |
| Birthweight centile <3 | 429/1,203 (35.7%; 33.0-38.4%) | 394/1,083 (36.4%; 33.6-39.3%) | 35/126 (27.8%; 20.7-36.2%) | | 0.027 |
| Birthweight centile 3 – 9.99 | 122/1,203 (10.1%; 8.6-12.0% ) | 114/1,083 (10.5%; 8.8-12.5%) | 8/126 (6.4%; 3.3-12.0%) | |  |
| Birthweight centile 10 – 90 | 548/1,203 (45.6%; 42.8-48.4%) | 478/1,083 (44.1%; 41.2-47.1%) | 70/126 (55.6%; 46.9-63.9%) | |  |
| Birthweight centile >90 | 104/1,203 (8.7%; 7.2-10.4%) | 97/1,083 (9.0%; 7.4-10.8%) | 7/126 (5.6%; 2.7-11.0%) | |  |
| Fetal growth restriction^a^ | 476/1,236 (38.5%; 35.8-41.3%) | 437/1,110 (39.4%; 36.5-42.3%) | 39/126 (31.0%; 23.5-39.5%) | | 0.066 |
| *Placental weight* | *312 (200-420)* | *311 (200-418)* | *336 (188-444)* | | *0.47* |
| Placental weight <10^th^ centile | 285/461 (61.8%; 57.3-66.2%) | 256/411 (62.3%; 57.5-66.8%) | 29/50 (58.0%; 44.2-70.6%)) | | 0.56 |
| Placental insufficiency^b^ | 446/602 (74.1%; 70.5-77.4%) | 403/538 (74.9%; 71.1-78.4%) | 43/64 (67.2%; 55.0-77.4%) | | 0.18 |

Individual cases (126/1,246) were excluded from accuracy assessment where either the actual documented or ideal causes of stillbirth (or both) were not reported and therefore could not be compared. Due to skewed distributions, continuous data are presented as median (interquartile range) and compared by Mann-Whitney u test. Categorical data are presented as number/total (percentage) and compared by chi squared test. ^a^ Fetal growth restriction was defined according to the Delphi consensus (Gordijn *et al* 2016). ^b^ Placental insufficiency was clinically defined as placental weight <10^th^ centile for sex/gestation, oligohydramnios in the absence of causative rupture of membranes or congenital abnormality, abnormal umbilical artery Doppler or contemporaneous description of an abnormally small, abnormal texture or abnormal shape placenta; placental histopathology or post-mortem examination findings were excluded from this contemporaneous clinical definition.

**Supplementary Table S4 Change in overall primary cause of death between actual Medical Certificate of Stillbirth and ideal Medical Certificate of Stillbirth after review of 1,246 cases**

| **ReCoDe domain/category** | **Actual MCS** | **Ideal MCS** |
| --- | --- | --- |
| **Fetal (All)** | **251**  **(20.1%; 18.0-22.5%)** | **494**  **(39.6%; 37.0-42.4%)** |
| (A1) Lethal congenital abnormality | 88 (7.1%; 5.8-8.6%) | 60 (4.8%; 3.8-6.2%) |
| (A2.1) Chronic fetal infection | 2 (0.2%; 0.0-0.6%) | 0 (0.0%; 0.0-0.3%) |
| (A2.2.) Acute fetal infection | 3 (0.2%; 0.1-0.7%) | 0 (0.0%; 0.0-0.3%) |
| (A3) Non-immune hydrops | 23 (1.9%; 1.2-2.8%) | 40 (3.2%;2.4-4.3%) |
| (A4) Iso-immunisation | 0 (0.0%; 0.0-0.3%) | 2 (0.2%; 0.0-0.6%) |
| (A5) Fetomaternal haemorrhage | 4 (0.3%; 0.1-0.8%) | 11 (0.9%; 0.5-1.6%) |
| (A6) Twin to twin transfusion syndrome | 13 (1.0%; 0.6-1.8%) | 12 (1.0%; 0.6-1.7%) |
| (A7) Fetal growth restriction | 49 (3.9%; 3.0-5.2%) | 306 (24.6%; 22.2-27.0%) |
| (A8) Fetal other | 69 (5.5%; 4.4-7.0%) | 63 (5.1%; 4.0-6.4%) |
| **Umbilical cord** | **7 (0.6%; 0.3-1.2%)** | **45 (3.6%; 2.7-4.8%)** |
| (B1) Umbilical cord prolapse | 1 (0.1%; 0.0-0.5%) | 6 (0.5%; 0.2-1.0%) |
| (B2) Constricting knot or loop of cord | 5 (0.4%; 0.2-0.9%) | 31 (2.5%; 1.8-3.5%) |
| (B3) Velamentous cord insertion | 0 (0.0%; 0.0-0.3%) | 5 (0.4%; 0.2-0.9%) |
| (B4) Umbilical cord other | 1 (0.1%; 0.0-0.5%) | 3 (0.2%; 0.1-0.7%) |
| **Placental** | **73 (5.9%; 4.7-7.3%)** | **238 (19.1%; 17.0-21.4%)** |
| (C1) Placental abruption | 58 (4.7%; 3.6-6.0%) | 146 (11.7%; 10.1-13.6%) |
| (C2) Placenta praevia | 0 (0.0%; 0.0-0.3%) | 1 (0.1%; 0.0-0.5%) |
| (C3) Vasa praevia | 0 (0.0%; 0.0-0.3%) | 0 (0.0%; 0.0-0.3%) |
| (C4) Placental insufficiency | 6 (0.5%; 0.2-1.0%) | 87 (7.0%; 5.7-8.5%) |
| (C5) Placental other | 9 (0.7%; 0.4-1.4%) | 4 (0.3%; 0.1-0.8%) |
| **Amniotic Fluid** | **17 (1.4%; 0.9-2.2%)** | **59 (1.7%; 3.7-6.1%)** |
| (D1) Chorioamnionitis | 9 (0.7%; 0.4-1.4%) | 47 (3.8%; 2.9-5.0%) |
| (D2) Oligohydramnios | 3 (0.2%; 0.1-0.7%) | 4 (0.3%; 0.1-0.8%) |
| (D3) Polyhydramnios | 1 (0.1%; 0.0-0.5%) | 8 (0.6%; 0.3-1.3%) |
| (D4) Amniotic fluid other | 4 (0.3%; 0.1-0.8%) | 0 (0.0%; 0.0-0.3%) |
| **Uterine** | **4 (0.3%; 0.1-0.8%)** | **8 (0.6%; 0.3-1.3%)** |
| (E1) Uterine rupture | 3 (0.2%; 0.1-0.7%) | 8 (0.6%; 0.3-1.3%) |
| (E2) Uterine other | 1 (0.1%; 0.0-0.5%) | 0 (0.0%; 0.0-0.3%) |
| **Maternal** | **39 (3.1%; 2.3-4.3%)** | **46 (3.7%; 2.8-4.9%)** |
| (F1) Diabetes | 16 (1.3%; 0.8-2.1%) | 26 (2.1%; 1.4-3.0%) |
| (F2) Thyroid disease | 0 (0.0%; 0.0-0.3%) | 3 (0.2%; 0.1-0.7%) |
| (F3) Pre-existing hypertension | 3 (0.2%; 0.1-0.7%) | 3 (0.2%; 0.1-0.7%) |
| (F4) Hypertensive disease in pregnancy | 10 (0.8%; 0.4-1.5%) | 8 (0.6%; 0.3-1.3%) |
| (F5) Lupus / Antiphospholipid syndrome | 0 (0.0%; 0.0-0.3%) | 0 (0.0%; 0.0-0.3%) |
| (F6) Cholestasis | 1 (0.1%; 0.0-0.5%) | 2 (0.2%; 0.0-0.6%) |
| (F7) Drug abuse | 0 (0.0%; 0.0-0.3%) | 2 (0.2%; 0.0-0.6%) |
| (F8) Maternal other | 9 (0.7%; 0.4-1.4%) | 2 (0.2%; 0.0-0.6%) |
| **Intrapartum** | **46 (3.7%; 2.8-4.9%)** | **8 (0.6%; 0.3-1.3%)** |
| (G1) Asphyxia | 46 (3.7%; 2.8-4.9%) | 8 (0.6%; 0.3-1.3%) |
| (G2) Birth trauma | 0 (0.0%; 0.0-0.3%) | 0 (0.0%; 0.0-0.3%) |
| **Traumatic** | **86 (6.9%; 5.6-8.4%)** | **152 (12.2%; 10.5-14.1%)** |
| (H1) External | 1 (0.1%; 0.0-0.5%) | 0 (0.0%; 0.0-0.3%) |
| (H2) Iatrogenic | 85 (6.8%; 5.6-8.4%) | 152 (12.2%; 10.5-14.1%) |
| **Unclassified** | **540 (43.3%; 40.6-46.1%)** | **151 (12.1%; 0.10-14.1%)** |
| (I1) No relevant condition identified | 540 (43.3%; 40.6-46.1%) | 150 (12.0%; 10.4-14.0%) |
| (I2) No information available | 0 (0.0%; 0.0-0.3%) | 1 (0.1%; 0.0-0.5%) |
| **No certificate should be issued** | **N/A** | **29 (2.3%; 1.6-3.3%)** |
| <24 weeks gestation | N/A | 24 (1.9%; 1.3-2.9%) |
| Neonatal death | N/A | 5 (0.4%; 0.2-0.9%) |
| ***Unable to assess*** | ***183 (14.7%; 12.8-16.8%)*** | ***16 (1.3%; 0.8-2.1%)*** |

The primary relevant condition at death was coded according to the Relevant Condition at Death (ReCoDe) classification system (Gardosi et al. 2005) according to the documentation on the actual Medical Certificate of Stillbirth (MCS) issued to the parents, and according to the local data collector-constructed ideal MCS after case note review. Data are expressed as number (percentage of the total included cohort) with 95% confidence interval. Bold text indicates the values for each overall domain of the ReCoDe classification, while body text of the table indicates the values for specific conditions within those domains.

**Supplementary Table S5: Frequency and accuracy of documentation/non-documentation of individual relevant conditions at death categories**

|  | **Documented (n=1,063)** |  | **Missed (n=1,032)** |  | |
| --- | --- | --- | --- | --- | --- |
|  |  | Of which, excluded after case review |  | Of which, originally designated “unexplained” | |
| **Fetal** | | | | |  |
| (A1) Lethal congenital abnormality | 122/1,063 (11.5%; 9.7-13.5%) | 27/113 (23.9%; 17.0-32.5%) | 12/1,032 (1.2%; 0.7-2.0%) | 1/12 (8.3%; 1.5-35.4%) | |
| (A2.1) Chronic fetal infection | 6/1,063 (0.6%; 0.3-1.2%) | 0/6 (0.0%; 0.0-39.0%) | 0/1,032 (0.0%; 0.0-0.4%) | - | |
| (A2.2.) Acute fetal infection | 4/1,063 (0.4%; 0.2-1.0%) | 4/4 (100.0%; 51.0-100.0%) | 1/1,032 (0.1%; 0.0-0.6%) | 1/1 (100%; 20.7-100%) | |
| (A3) Non-immune hydrops | 34/1,063 (3.2%; 2.3-4.4%) | 3/32 (9.4%; 3.2-24.2%) | 13/1,032 (1.3%; 0.7-2.1%) | 5/13 (38.5%; 17.7-66.5%) | |
| (A4) Iso-immunisation | 0/1,063 (0.0%; 0.0-0.4%) | N/A | 1/1,032 (0.1%; 0.0-0.6%) | 1/1 (100%; 20.7-100%) | |
| (A5) Fetomaternal haemorrhage | 4/1,063 (0.4%; 0.2-1.0%) | 2/4 (50.0%; 15.0-85.0%) | 4/1,032 (1.3%; 0.7-2.1%) | 4/4 (100%; 51.0-100%) | |
| (A6) Twin to twin transfusion syndrome | 15/1,063 (1.4%; 0.9-2.3%) | 0/13 (0.0%; 0.0-22.8%) | 4/1,032 (1.3%; 0.7-2.1%) | 1/4 (25%; 4.6-70.0%) | |
| (A7) Fetal growth restriction | 73/1,063 (6.9; 5.5-8.6%) | 20/73 (27.4%; 18.5-38.6%) | 265/1,032 (25.7%; 23.1-28.4%) | 195/265 (73.6%; 68.0-78.5%) | |
| (A8) Fetal other | 182/1,063 (17.1%; 15.0-19.5%) | 91/174 (52.3%; 44.9-59.6%) | 68/1,032 (6.6%; 5.2-8.3%) | 49/68 (72.1%; 60.4-81.3%) | |
| **Umbilical cord** | | | | |  |
| (B1) Umbilical cord prolapse | 3/1,063 (0.3%; 0.1-0.8%) | 3/3 (100.0%; 43.9-100.0%) | 2/1,032 (0.2%; 0.1-0.7%) | 2/2 (100%; 34.2-100%) | |
| (B2) Constricting knot or loop of cord | 8/1,063 (0.8%; 0.4-1.5%) | 2/8 (25.0%; 7.2-59.1%) | 20/1,032 (1.9%; 1.3-3.0%) | 16/20 (80.0%; 58.4-91.9%) | |
| (B3) Velamentous cord insertion | 0/1,063 (0.0%; 0.0-0.4%) | N/A | 4/1,032 (1.3%; 0.7-2.1%) | 3/4 (7.50%; 30.1-95.4%) | |
| (B4) Umbilical cord other | 3/1,063 (0.3%; 0.1-0.8%) | 3/3 (100.0%; 43.9-100.0%) | 5/1,032 (0.5%; 0.2-1.1%) | 4/5 (80.0%; 37.6-96.4%) | |
| **Placental** | | | | |  |
| (C1) Placental abruption | 76/1,063 (7.2%; 5.8-8.9%) | 2/76 (2.6%; 0.7-9.1%) | 55/1,032 (5.3%; 4.1-6.9%) | 34/55 (61.8%; 48.6-73.5%) | |
| (C2) Placenta praevia | 1/1,063 (0.1%; 0.0-0.5%) | 1/1 (100.0%; 20.7-100.0%) | 2/1,032 (0.2%; 0.1-0.7%) | 2/2 (100%; 34.2-100%) | |
| (C3) Vasa praevia | 0/1,063 (0.0%; 0.0-0.4%) | N/A | 1/1,032 (0.1%; 0.0-0.6%) | 0/1 (0.0%; 0.0-79.4%) | |
| (C4) Placental insufficiency | 12/1,063 (1.1%; 0.7-2.0%) | 2/12 (16.7%; 4.7-44.8%) | 277/1,032 (26.8%; 24.2-29.6%) | 184/277 (66.4%; 60.7-71.7%) | |
| (C5) Placental other | 12/1,063 (1.1%; 0.7-2.0%) | 12/12 (100.0%; 75.8-100.0%) | 7/1,032 (0.7%; 0.3-1.4%) | 4/7 (57.1%; 25.0-84.2%) | |
| **Amniotic fluid** | | | | |  |
| (D1) Chorioamnionitis | 10/1,063 (0.9%; 0.5-1.7%) | 1/8 (12.5%; 2.2-47.1%) | 44/1,032 (4.3%; 3.2-5.7%) | 29/44 (65.9%; 51.1-78.1%) | |
| (D2) Oligohydramnios | 14/1,063 (1.3%; 0.8-2.2%) | 13/14 (92.9%; 68.5-98.7%) | 7/1,032 (0.7%; 0.3-1.4%) | 3/7 (42.9%; 15.8-75.0%) | |
| (D3) Polyhydramnios | 1/1,063 (0.1%; 0.0-0.5%) | 0/1 (0.0%; 0.0-79.4%) | 30/1,032 (2.9%; 2.1-4.1%) | 17/30 (56.7%; 39.2-72.6%) | |
| (D4) Amniotic fluid other | 14/1,063 (1.3%; 0.8-2.2%) | 7/14 (50.0%; 26.8-73.2%) | 28/1,032 (2.7%; 1.9-3.9%) | 14/28 (50.0%; 32.6-67.4%) | |
| **Uterine** | | | | |  |
| (E1) Uterine rupture | 6/1,063 (0.6%; 0.3-1.2%) | 0/6 (0.0%; 0.0-39.0%) | 1/1,032 (0.1%; 0.0-0.6%) | 1/1 (100%; 20.7-100%) | |
| (E2) Uterine other | 2/1,063 (0.2%; 0.1-0.7%) | 1/2 (50.0%; 9.5-90.6%) | 3/1,032 (0.3%; 0.1-0.9%) | 0/3 (0.0%; 0.0-56.2%) | |
| **Maternal** | | | | |  |
| (F1) Diabetes | 28/1,063 (2.6%; 1.8-3.8%) | 19/28 (67.9%; 49.3-82.1%) | 27/1,032 (2.6%; 1.8-3.8%) | 22/27 (81.5%; 63.3-91.8%) | |
| (F2) Thyroid disease | 0/1,063 (0.0%; 0.0-0.4%) | N/A | 7/1,032 (0.7%; 0.3-1.4%) | 6/7 (85.7%; 48.7-97.4%) | |
| (F3) Pre-existing hypertension | 9/1,063 (0.9%; 0.5-1.6%) | 7/8 (87.5%; 52.9-97.8%) | 11/1,032 (1.1%; 0.6-1.9%) | 6/11 (54.6%; 28.0-78.7%) | |
| (F4) Hypertensive disease in pregnancy | 38/1,063 (3.6%; 2.6-4.9%) | 17/37 (46.0%; 31.0-61.6%) | 40/1,032 (3.9%; 2.9-5.2%) | 29/40 (72.5%; 57.2-83.9%) | |
| (F5) Lupus / Antiphospholipid syndrome | 0/1,063 (0.0%; 0.0-0.4%) | N/A | 2/1,032 (0.2%; 0.1-0.7%) | 2/2 (100%; 34.2-100%) | |
| (F6) Cholestasis | 1/1,063 (0.1%; 0.0-0.5%) | 1/1 (100.0%; 20.7-100.0%) | 4/1,032 (1.3%; 0.7-2.1%) | 3/4 (7.50%; 30.1-95.4%) | |
| (F7) Drug abuse | 1/1,063 (0.1%; 0.0-0.5%) | 0/1 (0.0%; 0.0-79.4%) | 4/1,032 (1.3%; 0.7-2.1%) | 4/4 (100%; 51.0-100%) | |
| (F8) Maternal other | 27/1,063 (2.5%; 1.8-3.7%) | 22/26 (84.6%; 66.5-93.9%) | 17/1,032 (1.7%; 1.0-2.6%) | 9/17 (52.9%; 31.0-73.8%) | |
| **Intrapartum** | | | | |  |
| (G1) Asphyxia | 48/1,063 (4.5%; 2.4-5.9%) | 46/47 (97.8%; 88.9-99.6%) | 6/1,032 (0.6%; 0.3-1.3%) | 6/6 (100%; 61.0-100%) | |
| (G2) Birth trauma | 0/1,063 (0.0%; 0.0-0.4%) | N/A | 0/1,032 (0.0%; 0.0-0.4%) | N/A | |
| **Traumatic** | | | | |  |
| (H1) External | 0/1,063 (0.0%; 0.0-0.4%) | N/A | 2/1,032 (0.2%; 0.1-0.7%) | 1/2 (50.0%; 9.5-90.6%) | |
| (H2) Iatrogenic | 90/1,063 (8.5%; 6.9-10.3%) | 2/85 (2.4%; 0.7-8.2%) | 53/1,032 (5.1%; 4.0-6.7%) | 2/53 (3.8%; 1.0-12.8%) | |
| **Unclassified** | | | | |  |
| (I1) No relevant condition identified | 625/1,063 (58.8%; 55.8-61.7%) | 506/614 (82.4%; 79.2-85.2%) | 11/1,032 (1.1%; 0.6-1.9%) | N/A | |
| (I2) No information available | 0/1,063 (0.0%; 0.0-0.4%) | N/A | 1/1,032  (0.1%; 0.0-0.6%) | 1/1 (100%; 20.7-100%) | |

Demonstrating (from left to right) i) the frequency with which individual Relevant Condition at Death (ReCoDe) categories were documented as causes of death (COD), whether primary or contributing (but excluding non-causal associations), on the actual Medical Certificate of Stillbirth (MCS) and ii) the proportion of these which were incorrectly documented (either not present or deemed not causal by local data collectors after case note review), iii) the frequency with which an individual ReCoDe category was considered to be causally implicated in the infant’s death but was not reported on the actual MCS and iv) the proportion of cases in which the original MCS stated an unknown COD. Data are expressed as number (percentage; 95% confidence interval) as a proportion of i) all cases where an actual certificate COD was reported (N=1,063), ii) all cases originally identified, where a matching ideal COD assessment was reported (denominator varies as detailed in table), iii) all cases with matching actual and ideal COD assessments reported (N=1,032) and iv) missed cases originally reported as unexplained COD on the actual MCS (denominator varies).Page Break

**Table S6: Odds of major error according to different independent variables**

|  | Whole cohort  (N=1,120) | | | Sensitivity cohort  (N=181) | | |
| --- | --- | --- | --- | --- | --- | --- |
|  | Major errors per category /total records in category | Odds ratio | 95% CI | Major errors per category/total records in category | Odds ratio | 95% CI |
| **Administrative variables** | | | |  |  |  |
| Profession of individual completing certificate: | | | | | | |
| Midwife | 295/484 | 1.00 | Reference | 100/147 | 1.00 | Reference |
| Doctor | 90/165 | 0.68 | 0.45-1.04 | 22/32 | 1.02 | 0.44-2.37 |
| Unknown | 169/275 | 0.82 | 0.55-1.22 | 0/1 | Insufficient variance | |
| No response | 142/196 | 1.10 | 0.64-1.88 | 1/1 | Insufficient variance | |
| Seniority of individual completing certificate: | | | | | | |
| Junior grade | 175/281 | 1.00 | Reference | 71/101 | 1.00 | Reference |
| Senior grade | 123/217 | 0.79 | 0.51-1.22 | 37/56 | 0.82 | 0.41-1.66 |
| Unknown | 218/364 | 0.93 | 0.61-1.41 | 14/23 | 0.66 | 0.25-1.70 |
| No response | 180/258 | 0.98 | 0.59-1.63 | 1/1 | Insufficient variance | |
| Case note access: | | | | | | |
| Full access | 514/819 | 1.00 | Reference | 108/160 | 1.00 | Reference |
| Electronic access only | 182/299 | 0.74 | 0.51-1.07 | 15/21 | 1.20 | 0.44-3.28 |
| No access | 0/2 | 0.44 | 0.02-8.52 | 0/0 | Insufficient variance | |
| **Previous audit region^a^** | | | |  |  |  |
| No | 632/996 | 1.00 | Reference | 102/146 | 1.00 | Reference |
| Yes | 64/124 | 0.50 | 0.28-0.89 | 21/35 | 0.65 | 0.30-1.39 |
| **Maternal / baseline variables** | | | |  |  |  |
| Maternal age (years): | | | | | | |
| *Per 5 years* | *676/1,091* | *0.96* | *0.87-1.08* | *123/181* | *0.78* | *0.59-1.02* |
| <20 | 18/31 | 1.02 | 0.46-2.26 | 5/5 | Insufficient variance | |
| 20-34.9 | 478/771 | 1.00 | Reference | 88/128 | 1.00 | Reference |
| 35-39.9 | 137/210 | 1.28 | 0.89-1.83 | 24/37 | 0.84 | 0.39-1.82 |
| 40+ | 43/79 | 0.68 | 0.41-1.13 | 6/11 | 0.55 | 0.16-1.89 |
| No response | 20/29 | 1.14 | 0.39-3.35 | 0/0 | Insufficient variance | |
| **Maternal body mass index (kg/m^2^):** | | | | | | |
| *Per 5kg/m^2^* | *672/1,071* | *1.18* | *1.07-1.31* | *122/178* | *1.36* | *1.07-1.73* |
| <18.5 | 20/35 | 0.77 | 0.37-1.63 | 8/11 | 1.69 | 0.41-6.96 |
| 18.5-24.9 | 259/414 | 1.00 | Reference | 41/67 | 1.00 | Reference |
| 25-29.9 | 171/289 | 0.85 | 0.60-1.18 | 26/41 | 1.10 | 0.49-2.45 |
| 30-39.9 | 168/166 | 1.12 | 0.79-1.59 | 34/46 | 1.80 | 0.79-4.08 |
| 40+ | 54/67 | 2.73 | 1.34-5.53 | 13/13 | Insufficient variance | |
| No response | 24/49 | 0.66 | 0.35-1.26 | 1/3 | 0.32 | 0.03-3.67 |
| Ethnic category: | | | | | | |
| White | 498/801 | 1.00 | Reference | 91/131 | 1.00 | Reference |
| Black | 52/90 | 0.80 | 0.48-1.32 | 8/12 | 0.88 | 0.25-3.08 |
| Asian | 92/136 | 1.15 | 0.74-1.78 | 17/26 | 0.83 | 0.34-2.02 |
| Other | 46/85 | 0.87 | 0.53-1.44 | 7/12 | 0.62 | 0.18-2.06 |
| No response | 8/8 | Insufficient variance | | 0/0 | Insufficient variance | |
| Smoker: | | | | | | |
| No | 530/864 | 1.00 | Reference | 25/34 | 1.00 | Reference |
| Yes | 141/215 | 1.27 | 0.89-1.80 | 97/145 | 1.37 | 0.59-3.17 |
| No response | 25/41 | 1.15 | 0.56-2.39 | 1/2 | 0.49 | 0.03-8.08 |
| Alcohol: | | | | | | |
| No | 650/1,046 | 1.00 | Reference | 2/3 | 1.00 | Reference |
| Yes | 11/18 | 0.76 | 0.28-2.09 | 117/173 | 0.96 | 0.08-11.30 |
| No response | 35/56 | 1.19 | 0.63-2.23 | 4/5 | 1.92 | 0.21-17.68 |
| Recreational drug abuse: | | | | | | |
| No | 635/1,019 | 1.00 | Reference | 116/170 | 1.00 | Reference |
| Yes | 17/28 | 0.68 | 0.30-1.54 | 4/7 | 0.60 | 0.12-2.96 |
| No response | 44/73 | 0.91 | 0.52-1.59 | 0/0 | Insufficient variance | |
| Parity: | | | | | | |
| *Per birth >24 weeks* | *679/1,094* | *0.90* | *0.82-0.99* | *123/181* | *0.76* | *0.60-0.95* |
| Primiparous | 307/459 | 1.00 | Reference | 65/84 | 1.00 | Reference |
| Multiparous | 373/641 | 0.76 | 0.59-0.98 | 58/97 | 0.43 | 0.22-0.84 |
| No response | 16/20 | 1.55 | 0.51-4.71 | 0/0 | Insufficient variance | |
| Previous stillbirth: | | | | | | |
| *Per stillbirth* | 679/1,039 | 0.37 | 0.18-0.76 | 123/181 | 0.47 | 0.03-7.60 |
| No | 667/1,070 | 1.00 | Reference | 122/179 | 1.00 | Reference |
| Yes | 12/28 | 0.42 | 0.19-0.94 | 1/2 | 0.47 | 0.03-7.60 |
| No response | 17/22 | 2.11 | 0.48-9.31 | 0/0 | Insufficient variance | |
| Previous late miscarriage (18^+0^ – 23^+6)^: | | | | | | |
| *Per late miscarriage* | 678/1,097 | 0.75 | 0.37-1.51 | 123/181 | 2.44 | 0.42-14.19 |
| No | 652/1,046 | 1.00 | Reference | 118/175 | 1.00 | Reference |
| Yes | 26/51 | 0.52 | 0.28-0.95 | 5/6 | 2.51 | 0.27-23.28 |
| No response | 18/23 | 2.29 | 0.54-9.84 | 0/0 | Insufficient variance | |
| **Pregnancy / Antenatal variables** | | | |  |  |  |
| Number of fetuses: | | | | | | |
| *Per fetus* | *696/1,118* | *1.07* | *0.63-1.81* | *123/181* | *1.44* | *0.28-7.36* |
| Singleton | 651/1,051 | 1.00 | Reference | 117/173 | 1.00 | Reference |
| Twin | 42/63 | 1.10 | 0.61-1.96 | 6/8 | 1.44 | 0.28-7.34 |
| Triplet | 3/4 | 0.86 | 0.07-10.17 | 0/0 | Insufficient variance | |
| No response | 0/2 | Insufficient variance | | 0/0 | Insufficient variance | |
| Gestation at first antenatal contact: | | | | | | |
| *Per week* | *650/1,045* | *0.99* | *0.97-1.01* | *123/181* | *0.97* | *0.92-1.02* |
| <18 weeks | 587/941 | 1.00 | Reference | 110/158 | 1.00 | Reference |
| 18+ weeks | 76/124 | 0.92 | 0.60-1.40 | 13/23 | 0.57 | 0.23-1.38 |
| Unbooked | 1/1 | Insufficient variance | | 0/0 | Insufficient variance | |
| No response | 32/54 | 0.73 | 0.38-1.38 | 0/0 | Insufficient variance | |
| Initial level of care: | | | | | | |
| Midwife-led | 276/444 | 1.00 | Reference | 42/65 | 1.00 | Reference |
| Consultant-led | 358/567 | 0.94 | 0.69-1.27 | 55/71 | 1.91 | 0.88-4.16 |
| Specialist-led | 55/97 | 0.60 | 0.34-1.05 | 26/45 | 0.70 | 0.27-1.079 |
| None | 1/1 | Insufficient variance | | 0/0 | Insufficient variance | |
| No response | 6/11 | 1.16 | 0.28-4.74 | 0/0 | Insufficient variance | |
| Highest level of care: | | | | | | |
| Midwife-led | 41/64 | 1.00 | Reference | 8/11 | 1.00 | Reference |
| Consultant-led | 386/587 | 1.07 | 0.58-1.96 | 58/78 | 1.10 | 0.26-4.68 |
| Specialist-led | 265/461 | 0.60 | 0.32-1.11 | 57/35 | 0.61 | 0.15-2.47 |
| None | 1/1 | Insufficient variance | | 0/0 | Insufficient variance | |
| No response | 3/7 | 0.99 | 0.16-6.17 | 0/0 | Insufficient variance | |
| Escalation of care in pregnancy: | | | | | | |
| No | 354/561 | 1.00 | Reference | 81/116 | 1.00 | Reference |
| Yes | 335/548 | 0.94 | 0.71-1.24 | 42/65 | 0.79 | 0.41-1.51 |
| Unknown | 7/11 | 1.89 | 0.37-9.65 | 0/0 | Insufficient variance | |
| Antenatally detected congenital abnormality: | | | | | | |
| No | 551/828 | 1.00 | Reference | 26/48 | 1.00 | Reference |
| Yes | 143/289 | 0.37 | 0.27-0.50 | 97/133 | 0.44 | 0.22-0.87 |
| Unknown | 2/3 | 0.81 | 0.06-10.22 | 0/0 | Insufficient variance | |
| Antenatal detected lethal congenital abnormality: | | | | | | |
| No | 637/988 | 1.00 | Reference | 116/166 | 1.00 | Reference |
| Yes | 54/125 | 0.27 | 0.18-0.42 | 7/15 | 0.38 | 0.13-1.10 |
| Unknown | 5/7 | 1.23 | 0.22-6.94 | 0/0 | Insufficient variance | |
| Ultrasound scans: | | | | | | |
| *Per scan* | *695/1,118* | *1.00* | *0.91-1.10* | *123/181* | *1.03* | *0.81-1.31* |
| No scan | 323/488 | 1.00 | Reference | 54/83 | 1.00 | Reference |
| Any scan | 373/572 | 1.08 | 0.82-1.41 | 69/98 | 1.28 | 0.68-2.42 |
| Oligohydramnios: | | | | | | |
| No | 560/910 | 1.00 | Reference | 100/149 | 1.00 | Reference |
| Yes | 69/100 | 1.61 | 0.98-2.64 | 15/21 | 1.22 | 0.45-3.35 |
| No response | 67/110 | 1.52 | 0.93-2.49 | 8/11 | 1.30 | 0.33-5.14 |
| Abnormal umbilical artery Doppler: | | | | | | |
| No | 489/794 | 1.00 | Reference | 90/134 | 1.00 | Reference |
| Yes | 78/120 | 1.12 | 0.72-1.72 | 13/20 | 0.91 | 0.34-2.44 |
| No response | 129/206 | 1.48 | 1.01-2.17 | 20/27 | 1.40 | 0.55-3.55 |
| Abnormal other Doppler: | | | | | | |
| No | 497/799 | 1.00 | Reference | 88/133 | 1.00 | Reference |
| Yes | 45/76 | 0.91 | 0.55-1.53 | 10/17 | 0.73 | 0.26-2.05 |
| No response | 154/245 | 1.42 | 0.98-2.05 | 25/31 | 2.13 | 0.82-5.57 |
| Antenatal fetal growth assessment: | | | | | | |
| Normal/no growth concern | 278/464 | 1.00 | Reference | 59/88 | 1.00 | Reference |
| Suspected FGR | 154/241 | 1.24 | 0.87-1.76 | 30/42 | 1.23 | 0.55-2.74 |
| LGA/accelerative | 40/60 | 1.56 | 0.81-2.98 | 9/14 | 0.88 | 0.27-2.88 |
| Too early to assess | 112/163 | 1.50 | 0.99-2.28 | 14/23 | 0.76 | 0.30-1.97 |
| No response | 112/192 | 1.08 | 0.73-1.60 | 11/14 | 1.80 | 0.47-6.96 |
| Termination of pregnancy: | | | | | | |
| No | 627/913 | 1.00 | Reference | 110/158 | 1.00 | Reference |
| Yes | 69/147 | 0.36 | 0.25-0.52 | 13/23 | 0.56 | 0.22-1.40 |
| **Diagnosis / Birth variables** | | | |  |  |  |
| Infant sex: | | | | | | |
| Male | 359/566 | 1.00 | Reference | 68/96 | 1.00 | Reference |
| Female | 326/535 | 0.97 | 0.74-1.27 | 55/85 | 0.75 | 0.40-1.42 |
| Unable to identify | 8/15 | 0.59 | 0.19-1.81 | 0/0 | Insufficient variance | |
| No response | 3/4 | 1.63 | 0.15-17.55 | 0/0 | Insufficient variance | |
| Gestation at diagnosis of fetal death *in utero:* | | | | | | |
| *Per week* | *681/1,090* | *0.99* | *0.96-1.01* | *123/181* | *0.97* | *0.92-1.03* |
| <24^+0^ | 23/23 | Insufficient variance | | 5/5 | Insufficient variance | |
| 24^+0^-27^+6^ | 212/322 | 1.09 | 0.74-1.59 | 31/44 | 1.41 | 0.58-3.46 |
| 28^+0^-33^+6^ | 170/306 | 0.65 | 0.45-0.95 | 36/54 | 1.19 | 0.51-2.74 |
| 34^+0^-36^+6^ | 114/176 | 1.19 | 0.75-1.86 | 20/27 | 1.69 | 0.59-4.88 |
| 37^+0^-40^+6^ | 148/237 | 1.00 | Reference | 27/43 | 1.00 | Reference |
| ≥41^+0^ | 14/26 | 0.65 | 0.28-1.55 | 4/8 | 0.59 | 0.13-2.70 |
| No response | 15/30 | 0.80 | 0.34-1.87 | 0/0 | Insufficient variance | |
| Timing of diagnosis of fetal death *in utero:* | | | | | | |
| Antenatal | 620/997 | 1.00 | Reference | 113/167 | 1.00 | Reference |
| Intrapartum | 53/88 | 1.01 | 0.61-1.65 | 10/11 | 4.77 | 0.60-38.21 |
| Postpartum | 5/5 | Insufficient variance | | 3/3 | Insufficient variance | |
| Unknown | 12/21 | 0.67 | 0.27-1.67 | 0/0 | Insufficient variance | |
| No response | 6/9 | 3.42 | 0.40-29.18 | 0/0 | Insufficient variance | |
| Diagnosis to delivery interval: | | | | | | |
| *Per day* | *687/1,098* | *1.00* | *0.98-1.02* | *123/181* | *1.00* | *0.95-1.05* |
| ≤1 week | 14/25 | 1.00 | Reference | 122/177 | 1.00 | Reference |
| > 1 week | 14/25 | 0.62 | 0.27-1.45 | 1/4 | 0.15 | 0.01-1.49 |
| No response | 9/22 | 0.61 | 0.24-1.56 | 0/0 | Insufficient variance | |
| Birthweight: | | | | | | |
| *Per 100g* | *695/1,118* | *0.99* | *0.98-1.00* | *123/181* | *0.98* | *0.96-1.01* |
| <1500g | 352/554 | 1.07 | 0.77-1.48 | 54/81 | 1.40 | 0.68-2.89 |
| 1500-2499.9g | 168/269 | 1.03 | 0.71-1.51 | 36/46 | 2.52 | 1.03-6.17 |
| 2500-4000g | 169/281 | 1.00 | Reference | 30/51 | 1.00 | Reference |
| >4000g | 6/14 | 0.89 | 0.28-2.90 | 3/3 | Insufficient variance | |
| No response | 1/2 | Insufficient variance | | 0/0 | Insufficient variance | |
| Individualised birthweight centile: | | | | | | |
| *Per 5 centiles* | *673/1,060* | *0.97* | *0.95-0.99* | *122/180* | *0.95* | *0.90-1.00* |
| <3 | 282/394 | 2.21 | 1.62-3.01 | 48/64 | 2.22 | 1.08-4.59 |
| 3 – 9.99 | 74/114 | 1.61 | 1.01-2.55 | 13/17 | 2.40 | 0.72-8.04 |
| 10 – 90 | 255/478 | 1.00 | Reference | 49/85 | 1.00 | Reference |
| >90 | 62/97 | 1.50 | 0.92-2.44 | 12/14 | 4.45 | 0.93-21.38 |
| No response | 23/37 | 1.44 | 0.65-3.17 | 1/1 | Insufficient variance | |
| Fetal growth restriction confirmed at birth^b^: | | | | | | |
| No | 372/673 | 1.00 | Reference | 65/106 | 1.00 | Reference |
| Yes | 317/437 | 2.22 | 1.67-2.96 | 58/75 | 2.19 | 1.10-4.35 |
| Unknown | 7/10 | 3.16 | 0.63-15.94 | 0/0 | Insufficient variance | |
| Placental weight: | | | | | | |
| *Per 50g* | *270/414* | *0.93* | *0.86-0.99* | *49/72* | *1.00* | *0.86-1.17* |
| <10^th^ centile^c^ | 182/256 | 2.12 | 1.33-3.37 | 31/43 | 1.58 | 0.58-4.31 |
| 10+ centile^c^ | 85/155 | 1.00 | Reference | 18/29 | 1.00 | Reference |
| No response | 429/709 | 1.08 | 0.71-1.64 | 74/109 | 1.29 | 0.55-3.03 |
| Placental insufficiency^d^: | | | | | | |
| No | 36/66 | 1.00 | Reference | 69/104 | 1.00 | Reference |
| Yes | 282/403 | 1.23 | 0.65-2.29 | 51/72 | 1.65 | 0.24-11.18 |
| Unknown | 378/651 | 0.64 | 0.35-1.16 | 3/5 | 1.33 | 0.20-9.70 |

Displaying the adjusted odds ratios for major error (defined as that which is likely to alter the interpretation of the Medical Certificate of Stillbirth by family, healthcare professionals or healthcare statisticians) by variable assessed within the whole accuracy cohort and the sensitivity cohort (those records with complete accuracy assessment submitted for sex, date, gestation, weight, timing in relation to labour and primary cause of death). All odds ratios are adjusted for clustering within hospitals. For each variable/category the number of records with major error and total number of records assessed is displayed along with the odds ratio (OR) and its 95% confidence interval. Due to missing data and small numbers of records in certain categories, particularly within the sensitivity cohort analyses, caution should be applied when interpreting OR from less frequently reported variables/categories. The grade of the completing clinician was determined as follows: junior (Registered Midwife band 5/6, Obstetrics and Gynaecology speciality trainee years 1-5 or equivalent), , or senior (Registered Midwife band 7+, Obstetrics and Gynaecology speciality trainee years 6+, Consultant Obstetrician or equivalent). Late miscarriage was defined as spontaneous miscarriage or fetal death *in utero* between 18^+0^ – 23^+6^ weeks gestation. Lethal congenital abnormalities were defined as those with a known >50% association with perinatal death. Oligohydramnios, abnormal umbilical artery Doppler and abnormal other Doppler (including but not limited to uterine artery, middle cerebral artery, Ductus venosus) were defined by local clinical diagnostic criteria. Customised estimated fetal weight and birthweight centiles were calculated using Bulk Centile Calculator v8.0.4 (Perinatal Institute, Birmingham UK). Abdominal circumference centiles were calculated according to Chitty et al 1994. ^a^ Hospital in previous audit region indicates a hospital that participated in the two previous audits of MCS accuracy in North West England (Cockerill et al. 2012, Higgins et al. 2018). ^b^ Fetal growth restriction was defined according to the Delphi consensus definition by Gordijn *et al.* 2016, with the additional inclusion criteria of loss of >50 centiles in symphysiofundal height in the absence of serial growth scans (provided that birthweight was below at least 50^th^ centile). ^c^ Gestation and sex adjusted placental weight centiles were calculated according to Thompson *et al*. 2007. ^d^ placental insufficiency was defined as the presence of any of the following i) placental weight <10^th^ centile for gestation/sex, ii) abnormal umbilical artery Doppler impedance, iii) abnormal uterine artery Doppler impedance, iv) oligohydramnios in the absence of causative rupture of membranes or fetal abnormality, v) clinician report of an abnormally small, abnormal texture or abnormal morphology placenta.
