# Supplementary material for: Worth the paper it’s written on? A cross-sectional study of Medical Certificate of Stillbirth accuracy in the UK
Source: Int J Epidemiol. 2022 Jun 20;52(1):295–308. doi: 10.1093/ije/dyac100 (PMC9908049; doi:10.1093/ije/dyac100)
Supplement: dyac100_Supplementary_Data [file dyac100_supplementary_data.zip › dyac100_Supplementary_Data/ije-2021-09-1359-File008.docx]

# Author contributions

MPR tested the data collection tool, directed the study’s implementation, contributed to the analytical strategy, helped to interpret the findings and edited the initial manuscript.

IH helped to interpret the findings and edited the manuscript.

WPS tested the data collection tool and edited the manuscript.

OR performed data collection (see statement in relation to NESTT working group members), and edited the manuscript.

JT reviewed the study protocol and edited the manuscript.

AEPH contributed to study design, helped interpret the findings and edited the manuscript.

LEH designed the study, wrote the study protocol, directed the study’s implementation, designed the analytical strategy, interpreted the findings and drafted the intial and edited manuscripts.

The NESTT working group members (HA, KFA, SA, EJB, LEB, LMB, MB, NB, REB, SAB, CBB, CEB, SHB, VB, CCC, SAC, FC-Ramsden, VCC, SC, LCC, AFC, JAD, DVD, CED, JKE, CIE, CHJE, DF, DMG-B, TG, SEG, EAG, JKG, MG, RG, EAG, AJH, RCH, JLH, FSH, EH, HSJ, LACJ, SJ, GJ, CMJ, IK, BK, LSK, RK, AJK, AK, CLBL, KRL, EAL, CAM, LRM, SMM, LMS, AM, KLM, AM, ESM, SVIM, OMM, HEM, SSM, MM, HM, LLM, KN, AEN, SKN, SOB, MHO, RIO, OO, NO, LP, MDP, MP, SRP, CLP, HMP, SEP, LP, OR, MR, KMR, NR, JMR, DRR, PS, FS, MS, KES, JKS-B, LJS, LIS, LJS, KS, CS, CJT, ST, KCAT, FT, JT, NV, HEW, KMW, SGW, KJW, SFW, WPW-T, CLMW) performed the data collection.
